# Stiffness reprogrammable magnetorheological metamaterials inspired by spine for multibit visual mechanical information processing

**DOI:** 10.1126/sciadv.ady8430

**Published:** 2025-10-08

**Authors:** Congcong Lou, Xinyu Lian, Huaxia Deng, Bing Liu, Shilong Duan, Yunpu Zhao, Xinglong Gong

**Affiliations:** ^1^ CAS Key Laboratory of Mechanical Behavior and Design of Materials, Department of Modern Mechanics, University of Science and Technology of China, Hefei, Anhui 230027, PR China.; ^2^ State Key Laboratory of Nonlinear Mechanics, Institute of Mechanics, Chinese Academy of Science, Beijing 100190, PR China.; ^3^ State Key Laboratory of Fire Science, University of Science and Technology of China, Hefei, Anhui 230026, PR China.

## Abstract

Embedding information processing into mechanical metamaterials is conductive to constructing multifunctional mechanical systems, which has unique advantages to provide information processing platforms in extreme environments. However, achieving high-density, reprogrammable, and visually readable information processing in most mechanical metamaterials remains a challenge. Here, we report a multibit programming spine structure strategy to create a magnetorheological metamaterial with high-density, reprogrammable, and visually readable information encoding capacities. Inspired by spine features, the magnetorheological spine beams, exhibiting substantial stiffness variation by bistable transition, meticulously conceived the stiffness reprogrammable magnetorheological metamaterial (SRMM). The SRMM exhibits a large stiffness conversion capability (40-fold) and high-density information encoding performance (10-bit). Coupling with the mechanoluminescent materials, the mechanical information achieves visualization conveniently, which is attributed to the conversion of the stiffness data into optical signals through optical energy level transitions. Such stiffness-based magnetorheological metamaterial offers expansive information encoding spaces, stable operation capabilities, and convenient readout approaches, advancing mechanical information processing system design for extreme environments.

## Teaser

A spine inspired stiffness-based MR metamaterial achieves multibit mechanical information processing and visual readout.

## INTRODUCTION

Mechanical metamaterials have opened innovative pathways for robust mechanical information transmission in extreme environments. Recently, mechanical information metamaterials (MIMs) have been developed on the basis of deformable multistable architectures (*1*–*4*), establishing programmable platforms for mechanical information operations. These systems leverage structural interactions to exhibit exceptional physical properties, including customized optical (*5*–*7*), acoustic (*8*, *9*), and mechanical behaviors (*10*, *11*), thereby allowing advanced information processing. Despite these advances, constructing MIMs with reconfigurable high-density information processing capabilities remains a critical challenge. These qualities make MIMs ideal for driving innovations in intelligent mechanical technologies, from reprogrammable displays (*12*, *13*) and mechanical computing architectures (*14*, *15*) to adaptive robotic systems (*16*, *17*).

Most existing MIMs are based on structural deformation of cells for encoding and computation (*18*–*21*). These fundamental cells are established on the basis of deformable structures, such as constrained beams (*22*–*24*), origami/kirigami architectures (*25*–*28*), curved shells (*29*–*31*), and composite spring systems (*32*). There is a huge potential energy difference between the two states of the cells, and through external stimuli, the state of units can be changed from the high-potential energy state to the lower state, showing a drop in altitude (*33*–*35*). However, it is difficult for the unit to switch from the lower state to the high state, which limits the reversible information programming for deformation-based MIMs. In addition, the information encoded by altitude is three-dimensional (3D), which is more difficult to read compared to 2D information. Unlike deformation-encoded systems, stiffness-programmable metamaterials offer intrinsic advantages in mechanical information processing due to their enhanced reconfigurability and state stability. Magnetorheological materials (*10*, *36*) demonstrate particular promise through field-responsive stiffness modulation, enabling the development of reprogrammable architectures with potential for high-density mechanical memory. However, three fundamental challenges impede practical implementation: (i) Traditional continuous stiffness adjustment via magnetorheological effects fails to ensure reliable state discrimination, necessitating discrete multistable switching mechanisms; (ii) mechanical states must maintain geometric invariance (constant structural height) across stiffness transitions to permit accurate information retrieval; (iii) the inherent opacity of stiffness variations demands innovative visualization strategies. Thus, amplifying the stiffness contrast between the two states and simplifying the process of stiffness identification are pivotal for advancing stiffness-based MIMs.

Here, we propose a multibit programmable spine structure strategy, constructing a stiffness reprogrammable magnetorheological metamaterial (SRMM) to enable high-density, reprogrammable, and visually readable mechanical information processing. Drawing inspiration from the anisotropic biomechanics of spine architectures, this paper achieves substantial stiffness variation (40-fold) and discrete stiffness switching (10-bit) by incorporating a specific gap arrangement in the structure, which, in combination with magnetic control, allows for the discrete switching between two stable stiffness states akin to the way the spine transitions between different bending states. We realize the high-density information encoding by integrating multiple parallel cells, with reprogrammable stiffness information visualized through the incorporation of mechanoluminescent materials. Theoretically, the bit capacity of multiple parallel cells can be defined by “*n* × *n* + 1.” Furthermore, leveraging the reprogrammable stiffness of each cell, logical operations are executed through coupled magnetic control. This advanced SRMM framework holds promise for robust information encoding and computational functions, advancing the development of intelligent platforms for mechanical information processing.

## RESULTS

### Construction of the SRMM

**Fig. 1.**
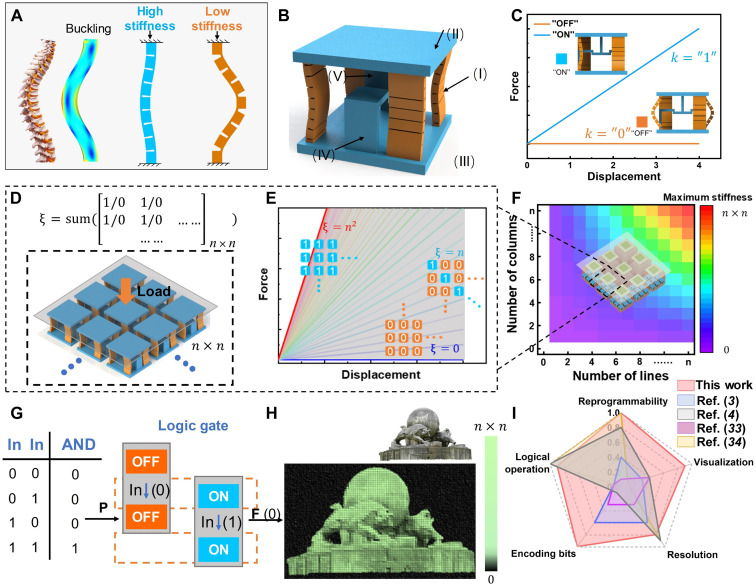
Design and programming of SRMM. (**A**) Structural design of MRS beam. (**B**) Schematic diagram of the SRMM cell, (I) MRS beams, (II) the top cover, (III) the base, (IV) T-shaped, and (V) L-shaped constraints. (**C**) The force-displacement relationship and the schematic diagram of the cell in ON and OFF states. (**D**) Array structure and stiffness programming method of SRMM. ξ represents the number of ON state cells in the array. (**E**) Reprogrammable force-displacement curves of SRMM. (**F**) The relationship between the maximum stiffness of the SRMM and the number of cells in array. (**G**) The calculation of AND gate logic in SRMM based on magnetic coupling control [input (0 1), output (0)]. (**H**) The encoding multibit information by parallel multiple cells in SRMM. (**I**) Comparison of the comprehensive properties of MIMs in the present work and published work in the radar map. The five parameters of comprehensive normalization include reprogrammability, visualization, resolution, encoding bits, and logical operation.

The stiffness variation capability between positive and quasi-zero stiffness states is fundamental for the design of the SRMM cells. Inspired by the spine structure, we develop magnetorheological spine (MRS) beams with magnetically reprogrammable stiffness through control of their bending direction. These MRS beams are fabricated by the hard magnetorheological elastomer (HMRE) powders through 3D printing, which require magnetization to achieve directional transition under a magnetic field. HMRE powders are mixtures of NdFeB particles and TPR 30A with the mass ratio of 1:1 (figs. S1 and S2). Then, the MRS beams are magnetized within a mold through exposure to a magnetic field of 1-T intensity for 15 min (fig. S10). Under magnetic fields applied in different directions, the MRS beams exhibit controllable bending switching properties. Figure 1A shows the ‌direction-dependent stiffness modulation of MRS beams‌ through embedding gaps in their tension-prone regions. Under unidirectional bending, these gaps ‌act as stress-relief channels‌, mitigating tensile strain and lowering the energy threshold for compression. Conversely, reversed bending ‌transforms the gaps into mechanical stoppers‌. Contact between gap edges under compressive stress restricts strain dissipation, thereby amplifying the external force required for deformation. This ‌bidirectional state transition effect, enabled by the gap’s dual role as a stress-release valve or stiffness-enhancing lock, demonstrates a programmable mechanical response, mirroring principles of spine structure.

The stable stiffness reprogramming requires the transition between discrete states. Magnetic morphing typically results in continuous curvature of the beams rather than discrete states. Here, we achieve bistability by designing a specific gap arrangement and overcoming the energy barrier through magnetic morphing, demonstrating an effective combination of mechanical bistability and magnetic control. The bistable cell composes of MRS beams and Polylactic Acid (PLA)-printed constraints. Four MRS beams are clamped to the top cover and bottom plate (Fig. 1B). A T-shaped constraint is attached to the top cover, while two L-shaped constraints are affixed to the bottom plate. These T-shaped and L-shaped constraints interlock, introducing an initial compression displacement to the MRS beams. The compression force-displacement curves of the bistable cell under different states are presented in Fig. 1C. In the “ON” state, the cell exhibits high stiffness, whereas in the “OFF” state, it demonstrates quasi-zero stiffness.

We establish the SRMM by periodically array of bistable cells (Fig. 1D), and the stiffness can be programmed by adjusting the number of cells in the ON state through the external magnetic field (Fig. 1E). Rather than a displacement-based metamaterial using serious collection, the proposed SRMM increases the bit capacity by the cells paralleling to achieve high-density information storage (Fig. 1F). Through the individual solenoid control, the SRMM realizes four different logical operations coupling with the magnetic control to simulate logical processing similar to electronic logic gates (Fig. 1G). Furthermore, the mechanical information is visualized by the prepared mechanoluminescent materials, translating the stiffness information to the optical signals. This visualization is convenient for the multibit information encoding, exempting as the “*n* × *n*” bits of the “Ruzi-Niu” Chart (Fig. 1H). The maximum stiffness of the multibit cell is determined by the number of cells, and the SRMM is composed of these multibit cells, demonstrating high-density and reprogrammable information processing capabilities. Illustrated in Fig. 1I, a radar plot shows the comparison between the SRMM and published works. Past studies predominantly focus on multibit mechanical information encoding via displacement data in series (*3*, *33*), often overlooking logical operations and facing limitations in reprogrammability. While Mei *et al.* (*4*) and Bilal *et al.* (*34*) establish binary logic platforms using vibration waves and parallel displacement data, this approach inherently restricts the bit capacity for information encoding. Unlike all prior work, our proposed metamaterial leverages a stiffness-based parallel mechanical information processing method, offering comprehensive advantages in reprogrammability, visualization, resolution, bit capacity, and logical functionality. In a word, the SRMM shows the potential to build multifunctional mechanical information processing platforms and provides a potential application possibility for stiffness-based mechanical metamaterials in the field of information processing.

### Mechanical properties of the MRS beam

**Fig. 2.**
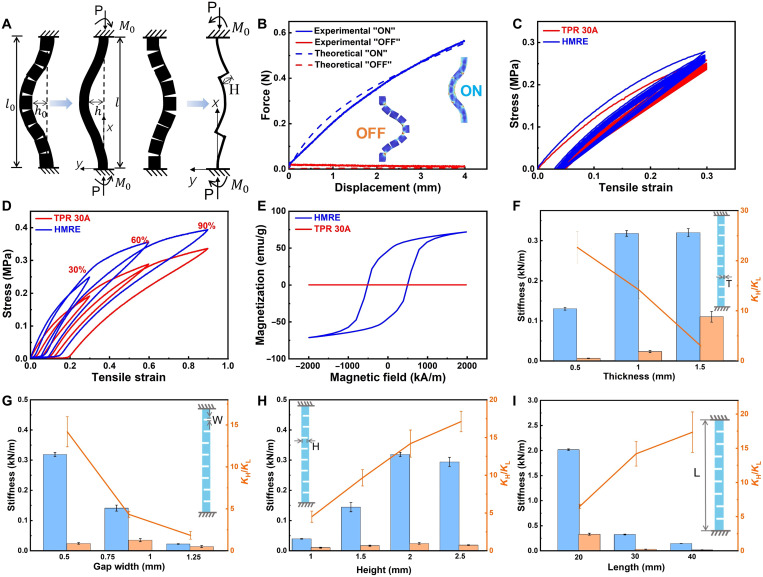
Mechanical properties of the printing materials and the impact of structural parameters on stiffness modulation in the MRS beam. (**A**) Simplified model of the MRS beam in two states. (**B**) Compression results of the MRS beam in two states. (**C**) Cyclic tensile tests of TPR 30A and HMRE. (**D**) Tensile tests of TPR 30A and HMRE under different strains. (**E**) The hysteresis loop of the TPR 30A and HMRE. (**F** to **I**) Stiffness of MRS beam with different geometric parameters in bistable states (blue columns indicate the ON state, and orange columns indicate the OFF state). emu, electromagnetic unit.

The stiffness variation of the MRS beam between the two states arises from the design of structural gaps. We establish a simplified model to describe the MRS beam in two states (Fig. 2A). In the ON state, the sides of the gaps come into contact during compression, allowing the MRS beam effectively approximated as a solid curved beam, thereby requiring a higher external load for compression. In contrast, in the OFF state, the MRS beam behaves as an irregular thin-walled structure due to the gaps, substantially reducing the compressive load needed for the same displacement (section S8). The deflection profile of the bending MRS beam under axial load is approximated by a cosine function

$$y=h\left[1,-,\text{cos},\left(\frac{2\pi x}{l}\right)\right]$$ (1)

where *h* is the maximum deflection of the MRS beam during bending, and *l* represents the height of the beam. Since the total length $L_{0}$ of the beam is almost constant, $\oint y\;\left(x\right)\;ds=L_{0}$ , the relationship between h and l can be obtained

$$h=\sqrt{\frac{l\left(L_{0}-l\right)}{\pi^{2}}}$$ (2)

The initial shape of the MRS in the ON state is defined as $\;y=h_{0}\left(1-\frac{\text{cos}2\pi x}{L_{0}}\right)$ . The beam bends under the axial load, according to the equation of the flexural line $\frac{{\text{d}}^{2}w}{\text{d}x^{2}}=\frac{M}{\mathrm{EI}}$ , the relationship between the axial load $P_{\text{ON}}$ andl is obtained

$$P_{\text{ON}}=\frac{EI_{\text{ON}}\left(h-h_{0}\right){\left(\frac{2\pi }{l}\right)}^{2}}{h}$$ (3)

where E is the equivariant modulus of the material (section S9), $I_{\text{ON}}$ is the moment of inertia of the simplified beam in the ON state, and the compression displacement $d=L_{0}-l$ . As for the MRS beam in the OFF state, relationship between the axial load $P_{\text{OFF}}$ and l is derived as follows

$$P_{\text{OFF}}=\frac{EI_{\text{OFF}}\left(h-h_{0}\right){\left(\frac{2\pi }{l}\right)}^{2}}{h}$$ (4)

where $I_{\text{OFF}}$ is the moment of inertia of the simplified beam in the OFF state. Figure 2B presents the force-displacement curves for both theoretical calculations and experimental results of the MRS beam in the ON and OFF states, illustrating high stiffness (240 N/m) in the ON state and quasi-zero stiffness (7 N/m) in the OFF state. Figure 2C shows the tensile stress-strain curves of HMRE and its corresponding matrix (TPR 30A) under 30% tensile strain for 20 cycles. The plastic deformation behavior mainly occurs during the first loading cycle. After that, changes in the stress-strain curves gradually become smaller, indicating the decrease of the Mullins effect. The tensile modulus of the HMRE and TPR 30A are 1.6 and 1.3 MPa in the linear elastic region ( ε < 5%), respectively. Figure 2D shows the stress-strain curves under different strains (30, 60, and 90%). The elastic modulus and strength of the HMRE are enhanced relative to pure TPR, attributed to the reinforcing effect of the NdFeB fillers. Figure 2E shows the magnetization curves of HMRE and TPR 30A, with the saturation magnetizations of ~75 and 0 electromagnetic unit g^−1^, respectively.

The mechanical properties of MRS beam not only depend on the characteristics of materials but also rely on the structural parameters: thickness (*T*), gap width (*W*), gap height (*H*), and length (*L*). To study the effects of four parameters (*T*, *W*, *H*, and *L*) on the stiffness variation of MRS beams, we conduct a series of compression tests (figs. S4 to S7). $K_{H}/K_{L}$ is the stiffness ratio of the MRS beam between two stable states, representing the resolution of the stiffness programmability. The programmability resolution of MRS beam exhibits remarkable thickness-dependent relationship, with an 800% stiffness ratio enhancement as thickness reduces from 1.5 to 0.5 mm (Fig. 2F). This tunability stems from contact mechanics at the gap interfaces. Wider gaps diminish contact tightness in the ON state, causing notable stiffness reduction (Fig. 2G), while marginally affecting both states through beam elongation. Conversely, increased gap height enhances ON state stiffness via improved contact, leaving the OFF state unaffected (Fig. 2H). Beam length universally reduces stiffness in both states following Eq. 2 (Fig. 2I), demonstrating decoupled control over absolute stiffness and programmability through geometric parameters. Here, we select the following structural parameters: *L* = 30 mm, *T* = 0.5 mm, *G* = 0.5 mm, and *H* = 2 mm, achieving a stiffness ratio of 40-fold. This parameter adjustment approach enables effective tuning of the resolution for programmable stiffness (fig. S8).

### Bistable characteristics of the MRS beam

**Fig. 3.**
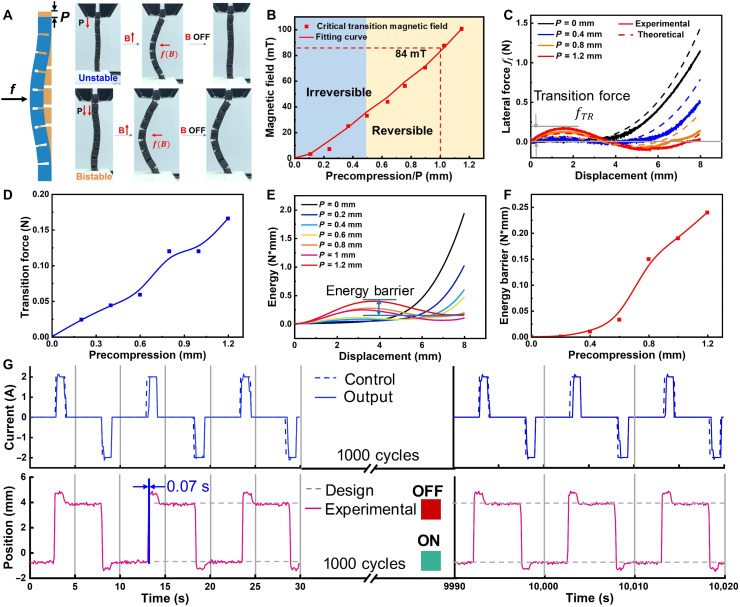
Bistable behavior of the MRS beam under precompression. (**A**) Diagram of the stability of MRS beam. (**B**) The relationship between the critical magnetic fields and precompression (irreversible/reversible switch). (**C**) The lateral force-displacement curves of MRS beam. (**D**) The transition force ( $f_{\text{TR}}$ ) in MRS beams. (**E**) The potential energy of the MRS beam. (**F**) The energy barrier heights of the MRS beam. (**G**) Reversible programming of more than 1000 cycles at 1-mm precompression displacement. The input current in the solenoid (top) and the corresponding central displacement of MRS beam generated by the magnetic field (bottom).

Initially, the MRS beam is in a stable ON state after applying precompression displacement *P* (Fig. 3A). The transition of two states in MRS beam is determined by external magnetic field. The transverse magnetic torque f(B) subjected to MRS beam is expressed as

$$f\left(B\right)=M\times B_{\text{app}}$$ (5)

where f(B) is the body torque generated by the magnetized domain under the applied magnetic field, M is the residual magnetization of the beam, and $B_{\text{app}}$ is the applied magnetic field density.

With a different precompression displacement (Fig. 3B), the MRS beam exhibits distinct transition modes controlled by the magnetic force f(B) . When the precompression displacement surpasses a critical threshold (0.5 mm) and the magnetic field beyond a critical transition value (84 mT for 1-mm precompression), the MRS beam can stably transition between the ON and OFF states under magnetic control. Otherwise, this transition cannot be reversibly controlled by transient magnetic field. The critical transition magnetic field increases with the precompression displacement. Furthermore, the relationship between the lateral force $f_{l}$ and the lateral displacement *d* is studied in detail by experiments and theoretical analysis (Fig. 3C). This theoretical relationship can be expressed as (section S10)

$$f_{l}=\frac{EI_{e}}{l^{3}}\frac{3\pi^{4}h^{2}d}{2t^{2}}\left[{\left(\frac{d}{h}-\frac{3}{2}\right)}^{2}-\left(\frac{1}{4}-\frac{4t^{2}}{3h^{2}}\right)\right]$$ (6)

where $I_{e}$ is the equivalent moment of inertia of the MRS beam, t is the thickness of the beam, *h* is the maximum deflection of the beam under compression, and l is the length of the beam under compression. The $f_{l}$ exhibits a cubic relationship with lateral displacement. The lateral force required for stable state transitions, termed the transition force$\;f_{\text{TR}}$ , corresponds to the first extremum in the force-displacement curve and is provided by the external magnetic field [ $f\left(B\right)>f_{\text{TR}}$ ]. Notably, this transition force increases with the precompression displacement (Fig. 3D).

The potential energy distribution along the displacement of the MRS beam is obtained by integrating the force-displacement curve. As illustrated in Fig. 3E, an increase in precompression displacement results in the formation of two distinct potential wells, which offers further clarification of the transition behavior observed in Fig. 3B. The transition involves overcoming the potential barrier, represented as the “hump” in the curve between the two wells. As the precompression displacement increases, the potential barrier between the two states rises, indicating an increase in the stability of the states (Fig. 3F).

To demonstrate the robust reversibility of the state transition, we conduct a 1000-cycle magnetic switching experiment on the MRS beam at 0.8-mm precompression displacement (Fig. 3G and fig. S9). The programmed power continuously supplies a steady current of 2 A. The input current is programmed by using a relay, delivering a 2-A current with a constant gradient lasting 1 s to switch the state of the beam. Each impulse is separated by a 4-s pause to avoid overheating of the electromagnet. During this pause, without the magnetic field, the MRS beam remains stable in either the ON or OFF state. A laser displacement sensor is used to measure the central position response of the MRS beam in periodic cycles, with data recorded by an oscilloscope. Subjected to the periodic pulsed current, the MRS beam exhibits a rapid state transition response (0.07 s). The MRS beam consistently reaches the designed ON and OFF position predictably, reproducibly, and without noticeable deterioration during the whole cyclic tests.

### Stiffness reprogrammable functions of the SRMM

**Fig. 4.**
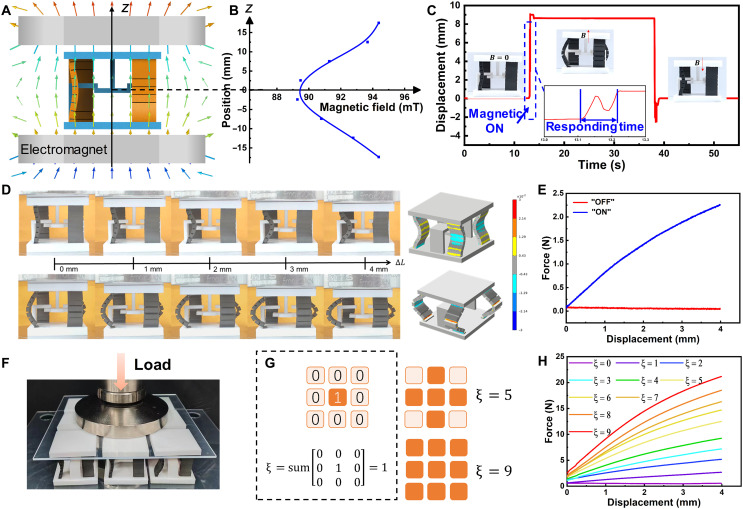
Stiffness reprogramming capability of the cell and SRMM. (**A**) The schematic of magnetic field application setup for cell. (**B**) The strength of the magnetic field at different positions. (**C**) The transition speed of the cell between two states under magnetic field. (**D**) Experiments and simulations of the cell under compression in two states. (**E**) The force-displacement curves of the cell in two states. (**F**) Experimental image of SRMM composed of 3 by 3 cells. (**G**) Stiffness programming mode of SRMM. (**H**) The force-displacement curves of SRMM under different stiffness programs.

The SRMM cell comprises four MRS beams and PLA-printed constraints (two L-shaped and one T-shaped), which induce precompression displacement, thereby enabling reversible switching (Fig. 4A). We place the cell in the magnetic field that generated by electromagnets. As shown in Fig. 4B, the magnetic field strength decreases with increasing distance from the magnet surfaces, reaching a minimum at the midpoint between the two magnets. While in the central region, where the MRS beam is positioned, the magnetic field remains relatively uniform, satisfying the conditions required for consistent state switching of the beam (89 mT). It is sufficient to meet the critical transition magnetic field (84 mT) requirements of the cell with the 1-mm precompression (figs. S11 and S12). By altering the direction of the applied magnetic field, the cells can be switched between two distinct states (ON and OFF states), with the transition occurring rapidly, typically within about 0.12 s (Fig. 4C and movie S1). We conduct experiments and finite element modeling simulations to examine the compressive deformation of the cells in both states, confirming the theoretical prediction (Fig. 4D). In the ON state, the gap sides come into contact under compression, resisting further deformation and resulting in a high-stiffness configuration. In the OFF state, only the thickness layer resists compression, leading to a lower stiffness (Fig. 4E).

The variation in stiffness of the cell between two distinct states reflects the system’s ability to distinguish and store different mechanical states. The stiffness variation determines the resolution of mechanical information storage. By enabling precise switching and differentiation between more states, the system’s storage density and response accuracy can be substantially enhanced.

The SRMM consists of arranging multiple cells, and we realize the stiffness programmable performance by using the magnetic field to regulate each cell separately. As illustrated in Fig. 4F, a “3 by 3” array of cells forms a typical SRMM. In this configuration, the stiffness programming of the SRMM is governed by the number of ON state cells ξ (Fig. 4G). It is worth noting that the stiffness of the SRMM is independent of the position of the cells in the ON state (fig. S13). The force-displacement curves of the SRMM with different ξ are presented in Fig. 4H. For a given compression displacement, the required external force increases with the ξ , indicating that the 3 by 3 array SRMM can achieve “10” distinct stiffness programs (bits). By combining different cells in various array configurations, it becomes possible to enable multibit controllable mechanical information processing within the SRMM.

### Information encoding and visualization of SRMM

**Fig. 5.**
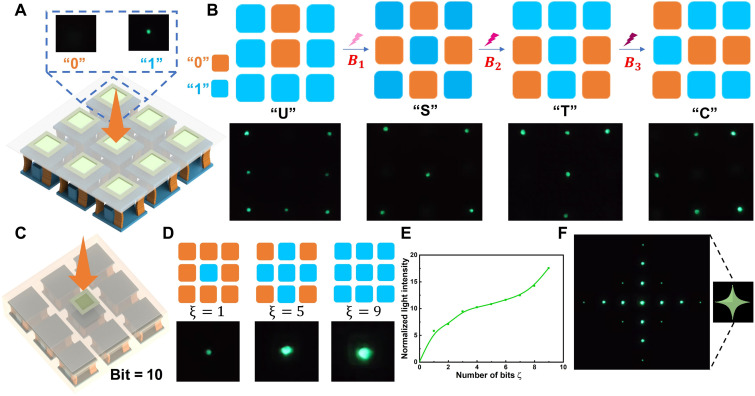
Encoding and visualization of SRMM (3 by 3 array). (**A**) The 2 bits visualized SRMM. (**B**) The 2 bits SRMM encodes “U,” “S,” “T,” and “C” sequentially under magnetic fields. (**C**) The 10-bit visualized SRMM unit. (**D**) Light forms corresponding to stiffness programs. (**E**) Relationship between light intensity and “ξ.” (**F**) Light spots of varying intensities form a star pattern.

We visualize the stiffness of SRMM by preparing mechanoluminescent materials integrated with cells (Fig. 5A and fig. S17). Stiffness information is directly read out through light intensity, offering a more intuitive means of identifying and displaying mechanical properties. The external loading is transferred to the mechanoluminescent material through the ON state cell, owing to its high stiffness, thereby inducing light emission. In contrast, when the cell is in the OFF state, its quasi-zero stiffness prevents the load from being transmitted to the mechanoluminescent material, resulting in darkness. Therefore, adjusting the state of the cells by programming magnetic fields and applying external loading, the stiffness information can be encoded in the light spot through magnetic control. As demonstrated in Fig. 5B, the light spots sequentially form the letters “U,” “S,” “T,” and “C” (movie S2).

To enable the encoding of pattern information, SRMMs with multibit programming capabilities are essential. Here, we form a 10-bit visualized SRMM unit by an array of nine cells (Fig. 5C). By adjusting the number of ON state cells within the unit, the stiffness of SRMM unit can be reprogrammed, thereby modulating the light intensity (Fig. 5, D and E, and figs. S15 and S16). This light modulation mechanism allows for the construction of 10-bit SRMMs. As shown in Fig. 5F, the 10-bit SRMM generates a “star” pattern. Furthermore, as the number of unit cells increases, more complex patterns can be encoded, highlighting the multibit SRMM’s capacity for high-density and readable information processing.

### Information logic computing capabilities of SRMM

**Fig. 6.**
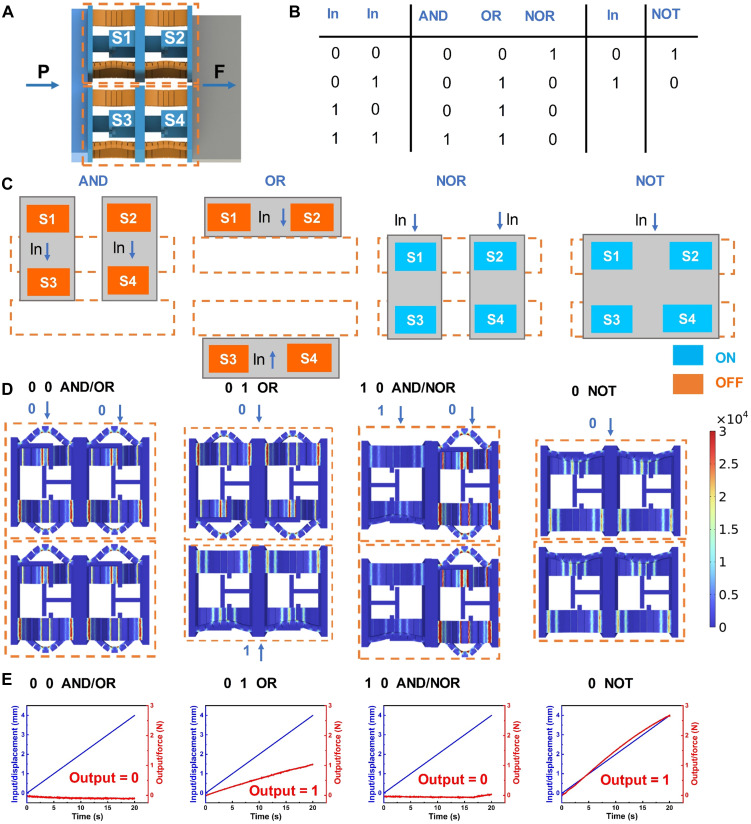
Information logic computation. (**A**) The mechanical logic gate formed by four cells. (**B**) Truth tables for different kinds of logic gates. (**C**) Coupling control of different mechanical logic operations and initial states. (**D**) Numerical simulation of the mechanical logic gate under different inputs and logic operations. (**E**) Inputs and outputs of different logical operations.

The highly programmable stiffness characteristics of the cells enable the implementation of mechanical logic operations. As illustrated in Fig. 6A, we realize the desired mechanical logic gate by connecting two parallel cells in series. This four-cell mechanical logic gate functions similarly to conventional electronic logic gates. For instance, in an “AND” logic operation, the two switches are arranged in series, such that the signal can only pass through if both switches are in the ON state. In other words, when both inputs are “1” (true), the output of the logic operation is also 1 (true). Conversely, when the switches are connected in parallel, the circuit allows signal output when either switch is ON, corresponding to an “OR” gate (Fig. 6B).

To realize all logical gates, we use a configuration of four interconnected switches. An input of “0” signifies that the initial state of the cells remains unchanged, while an input of 1 triggers a change in the cells’ state. For example, to implement the AND gate, the states of cells S1 and S3 represent one input, and the states of cells S2 and S4 represent another binary input. Figure 6C shows the initial state of the AND gate, where both inputs are 0, resulting in an output of 0. In the case of the OR gate, S1 and S2 are coupled, as are S3 and S4, with the output becoming true (1) if either input is 1 (fig. S18 and movie S3). The coupling configuration for the “NOR” gate mirrors that of the AND gate, but with a different initial state. For the “NOT” gate, all four cells are coupled, and all initial states are set to ON. Regarding the determination of binary outputs (0 or 1) based on the force-displacement response, we establish a quantitative threshold to classify the logic state. Specifically, if the output stiffness (slope of the force-displacement curve) exceeds three times the stiffness of a single cell in the OFF state, the output is classified as 1; otherwise, it is defined as 0. We simulate the stress distribution of the structure under different inputs for different logic gates, with the results presented in Fig. 6D. For instance, in the case of the AND and OR gates, when the inputs are 0 and 0, the cells in the mechanical logic gate remain in the OFF state. Upon applying displacement to the left side, the force output on the right side is quasi-zero (Fig. 6E). By combining these logical gates, mechanical logic operations with complex computational functions can potentially be realized.

## DISCUSSION

In summary, we proposed a multibit programming spine structure strategy to realize the high-density and reprogrammable mechanical information processing by constructing an SRMM. We explored its potential as a mechanical information processing platform through both experimental testing and concept demonstration. In contrast to metamaterials based on deformation for information processing, our platform relied on stiffness programming and visualization, presenting excellent information processing reprogrammability. The structural design of MRS beam in SRMM made its stiffness differ by an order of magnitude in different bending directions. The bistable properties of the MRS beam came from the constraints in the cell, and the bending direction can be programmed under the stimulation of the magnetic field. The SRMM composed of an array of cells, and the bistable states in the cells can be independently controlled, resulting in the stiffness of the SRMM can be reprogrammed.

In view of the independence of the cell stiffness regulation, we realized the writing and erasing of information by programming the stiffness of cells. The stiffness information was visualized by assembling the mechanoluminescent materials. The cells with different stiffness presented light spots with different light intensity under the external load. By replacing a single cell with a multibit cell, the number of cell bits increased from “2” to *n* × *n* + 1. In this way, the multibit high-density information encoding can be realized on the basis of the mechanical information platforms constructed by SRMM. Furthermore, with the recognition feature of whether stress propagation was allowed, the SRMM can perform different logic gate operations through magnetic coupling control. Although at present these demonstrations were not yet practical for large-scale data encoding, they provided a metamaterial perspective on information encoding and material design. With advances in 3D printing at the submicrometer scale and using multiple materials, the miniaturization needed for higher storage capacity may soon be within reach owing to the scale independence of the underlying physical mechanisms. The design strategy suggested pathways to creating additional stimulus-responsive materials that can achieve multifunction. In conclusion, the proposed stiffness-based SRMM provided a broad prospect for the development of mechanical metamaterials in an information processing platform.

## MATERIALS AND METHODS

### 3D printing

The preparation method for the powders used in printing was as follows (fig. S1): The TPR 30A particles were first frozen through liquid nitrogen and then quickly ground with an open mill. This step was repeated until the particles were ground to a powder with a diameter of less than 2 mm. Then, the TPR 30A (obtained from Hanwha TOTAL Petrochemical Co. Ltd.) powders were evenly mixed with the NdFeB particles (obtained from Guangzhou Nord Transmission Co. Ltd.) at 50°C, allowing the NdFeB particles to coat the surface of the TPR 30A powders. The mixed powders were then poured into the screw extruder to obtain the magnetic elastomer wires. Last, the magnetic wires were frozen in liquid nitrogen and ground to obtain the composite hard magnetic powders with a diameter of 2 mm. The MRS beam was prepared by a screw extrusion printing method. The nozzle diameter was 0.4 mm, the height of each layer was 0.25 mm, and the filling density was set to 100%. The print speed was 15 mm/s, and the nozzle temperature was set to 160°C.

The microstructures of the materials were observed by scanning electron microscopy. The microstructure of the NdFeB particles was shown in fig. S2, with an average particle size of about 7 μm. The microstructure of the NdFeB powders used in the final printing was shown in fig. S2, where the NdFeB particles were evenly distributed in the TPR 30A. The surface of the printed sample was smooth, and the printing precision was high. The uniform distribution of hard magnetic particles in the TPR matrix improved because of the heating and extrusion process in the nozzle.

### Mechanoluminescent material fabrication

ZnS:Cu particles were mixed with poly(dimethylsiloxane) (PDMS) in the ratio of 7:3 and formed a uniform dispersion followed by adding the curing agent according to a PDMS/curing agent ratio of 10:1. Then, SiO_2_ nanoparticles of 1.25% mass fraction of the total mixed solution were added and stirred for 5 min, and the mixed solution was put into a vacuum pump to evacuate the internal air bubbles. The above mixed solution was further added into the acrylonitrile butadiene styrene plastic mold and cured at 80°C for 30 min to obtain a mechanoluminescent layer (fig. S14).

### Finite element modeling

The simulations of the model in Figs. 4 and Fig. 6 and fig. S12 were calculated in commercial software COMSOL. The structural parameters of the MRS beam were as follows: length = 30 mm, width = 10 mm, thickness = 0.5 mm, and gap height = 2 mm. The Young’s modulus was 1.6 MPa, the density was 3000 kg/m^2^, and the Poisson’s ratio was 0.45. The deformation of the MRS beam after axial compression displacement of 1 mm was taken as the initial shape. To capture the intrinsic asymmetric response of MRS beams during deformation, we implemented clamped boundary conditions at both ends while establishing precise contact pair definitions along all gap interfaces. The residual magnetic flux density of the MRS beam was set at 5 mT, and an external magnetic field of 200 mT was applied. The MRS beam deformed under the Maxwell force.

### Mechanical experiments

The microstructures of the printed materials were observed using a scanning electron microscope (Gemini 500, Carl Zeiss Jena, Germany). The magnetic properties of samples were characterized using a Hysteresis Measurement of Soft and Hard Magnetic materials (HyMDC Metis, Leuven, Belgium). The tensile and compression performance of printed films and structures were obtained by a dynamic mechanical analyzer (ElectroForce 3200, TA Instruments, USA) and a universal tensile instrument (Criterion Model 43, MTS Co. Ltd., China) with a 500-N force sensor module. The loading rate was set at 0.1 mm/s.

## References

[1] H. Yasuda, P. R. Buskohl, A. Gillman, T. D. Murphey, S. Stepney, R. A. Vaia, J. R. Raney, Mechanical computing. Nature 598, 39–48 (2021). 34616053 10.1038/s41586-021-03623-y

[2] C. Coulais, E. Teomy, K. de Reus, Y. Shokef, M. Hecke, Combinatorial design of textured mechanical metamaterials. Nature 535, 529–532 (2016). 27466125 10.1038/nature18960

[3] Z. Meng, H. Yan, M. Liu, W. Qin, G. M. Genin, C. Q. Chen, Encoding and storage of information in mechanical metamaterials. Adv. Sci. 10, 2301581 (2023). 10.1002/advs.202301581 PMC10369242 37083263

[4] T. Mei, Z. Meng, K. Zhao, C. Q. Chen, A mechanical metamaterial with reprogrammable logical functions. Nat. Commun. 12, 7234 (2021). 34903754 10.1038/s41467-021-27608-7 PMC8668933

[5] M. B. Kristensen, N. Kralj, E. C. Langman, A. Schliesser, Long-lived and efficient optomechanical memory for light. Phys. Rev. Lett. 132, 100802 (2024). 38518344 10.1103/PhysRevLett.132.100802

[6] A. Calzolari, A. Catellani, M. B. Nardelli, M. Fornari, Hyperbolic metamaterials with extreme mechanical hardness. Adv. Opt. Mater. 9, 2001904 (2021).

[7] Z. Yan, A. D. Handoko, W. Wu, C. Yang, H. Wang, M. Yilmaz, Z. Zhang, L. Cheng, X. Cheng, G. W. Ho, B. Feng, N. Shibata, R. Zhao, J. K. W. Yang, C. T. Chong, Y. Ikuhara, C. W. Qiu, Atomic-engineered gradient tunable solid-state metamaterials. Proc. Natl. Acad. Sci. U.S.A. 121, e2408974121 (2024). 39292742 10.1073/pnas.2408974121 PMC11441542

[8] T. Frenzel, J. Köpfler, E. Jung, M. Kadic, M. Wegener, Ultrasound experiments on acoustical activity in chiral mechanical metamaterials. Nat. Commun. 10, 3384 (2019). 31358757 10.1038/s41467-019-11366-8 PMC6662661

[9] L. Wu, Y. Wang, K. Chuang, F. Wu, Q. Wang, W. Lin, H. Jiang, A brief review of dynamic mechanical metamaterials for mechanical energy manipulation. Mater. Today 44, 168–193 (2021).

[10] B. Zou, Z. Liang, D. Zhong, Z. Cui, K. Xiao, S. Shao, J. Ju, Magneto-thermomechanically reprogrammable mechanical metamaterials. Adv. Mater. 35, e2207349 (2023). 36385420 10.1002/adma.202207349

[11] S. Janbaz, K. Narooei, T. Manen, A. A. Zadpoor, Strain rate–dependent mechanical metamaterials. Sci. Adv. 6, eaba0616 (2020). 32596451 10.1126/sciadv.aba0616 PMC7299623

[12] C. Yue, W. Zhao, F. Li, B. Li, L. Liu, Y. Liu, J. Leng, A flexibly function-oriented assembly mechanical metamaterial. Adv. Funct. Mater. 34, 2316181 (2024).

[13] H. Y. Lee, M. Gu, J. Hwang, H. Hwang, Y. S. Kim, S. Y. Lee, S. H. Kim, Auxetic photonic patterns with ultrasensitive mechanochromism. Adv. Sci. 11, 2304022 (2024). 10.1002/advs.202304022 PMC10767460 37942590

[14] L. J. Kwakernaak, M. Hecke, Counting and sequential information processing in mechanical metamaterials. Phys. Rev. Lett. 130, 268204 (2023). 37450791 10.1103/PhysRevLett.130.268204

[15] X. Zheng, X. Zhang, T. T. Chen, I. Watanabe, Deep learning in mechanical metamaterials: From prediction and generation to inverse design. Adv. Mater. 35, e2302530 (2023). 37332101 10.1002/adma.202302530

[16] A. Rafsanjani, K. Bertoldi, A. R. Studart, Programming soft robots with flexible mechanical metamaterials. Sci. Robot. 4, eaav7874 (2019). 33137714 10.1126/scirobotics.aav7874

[17] X. Huang, W. Guo, S. Liu, Y. Li, Y. Qiu, H. Fang, G. Yang, K. Zhu, Z. Yin, Z. Li, H. Wu, Flexible mechanical metamaterials enabled electronic skin for real-time detection of unstable grasping in robotic manipulation. Adv. Funct. Mater. 32, 2109109 (2022).

[18] J. K. Choe, J. Yi, H. Jang, H. Won, S. Lee, H. Lee, Y. Jang, H. Song, J. Kim, Digital mechanical metamaterial: Encoding mechanical information with graphical stiffness pattern for adaptive soft machines. Adv. Mater. 36, e2304302 (2024). 37850948 10.1002/adma.202304302

[19] F. Pan, Y. Li, Z. Li, J. Yang, B. Liu, Y. Chen, 3D pixel mechanical metamaterials. Adv. Mater. 31, e1900548 (2019). 31074009 10.1002/adma.201900548

[20] Y. Yang, J. Feng, D. P. Holmes, Mechanical computing with transmissive snapping of kirigami shells. Adv. Funct. Mater. 34, 2403622 (2024).

[21] H. Yasuda, T. Tachi, M. Lee, J. Yang, Origami-based tunable truss structures for non-volatile mechanical memory operation. Nat. Commun. 8, 962 (2017). 29042544 10.1038/s41467-017-00670-w PMC5714951

[22] Y. Chi, Y. Li, Y. Zhao, Y. Hong, Y. Tang, J. Yin, Bistable and multistable actuators for soft robots: Structures, materials, and functionalities. Adv. Mater. 34, 2110384 (2022). 10.1002/adma.202110384 35172026

[23] Q. Bai, T. Zhou, C. Gan, Q. Wang, X. Zheng, K. X. Wei, A triboelectric-piezoelectric hybrid nanogenerator for rotational energy harvesting based on bistable cantilever beam. Energy Conver. Manage. 300, 117971 (2024).

[24] Y. Cao, M. Derakhshani, Y. Fang, G. Huang, C. Cao, Bistable structures for advanced functional systems. Adv. Funct. Mater. 31, 2106231 (2021).

[25] C. Huang, T. Tan, Z. Wang, X. Nie, S. Zhang, F. Yang, Z. Lin, B. Wang, Z. Yan, Bistable programmable origami based soft electricity generator with inter-well modulation. Nano Energy 103, 107775 (2022).

[26] H. Zhan, G. Zhang, J. M. Bell, V. B. C. Tan, Y. Gu, High density mechanical energy storage with carbon nanothread bundle. Nat. Commun. 11, 1905 (2020). 32312980 10.1038/s41467-020-15807-7 PMC7171126

[27] H. Fang, S. C. A. Chu, Y. Xia, K. W. Wang, Programmable self-locking origami mechanical metamaterials. Adv. Mater. 30, e1706311 (2018). 29513374 10.1002/adma.201706311

[28] S. Wu, Q. Ze, J. Dai, N. Udipi, G. H. Paulino, R. Zhao, Stretchable origami robotic arm with omnidirectional bending and twisting. Proc. Natl. Acad. Sci. U.S. A. 118, e2110023118 (2021). 34462360 10.1073/pnas.2110023118 PMC8433528

[29] J. Shi, H. Mofatteh, A. Mirabolghasemi, G. Desharnais, A. H. Akbarzadeh, Programmable multistable perforated shellular. Adv. Mater. 33, e2102423 (2021). 34467581 10.1002/adma.202102423

[30] Z. Chen, S. Kong, Y. He, S. Chen, W. Wang, L. Jin, S. Zhang, Y. Hong, L. Pan, H. Wu, Y. Xie, C. Linghu, Z. Mao, Z. Yang, C. H. Chan, J. Song, J. Lu, A magnet-driven soft bistable actuator. Adv. Funct. Mater. 34, 2311498 (2024).

[31] N. Vasios, B. Deng, B. Gorissen, K. Bertoldi, Universally bistable shells with nonzero Gaussian curvature for two-way transition waves. Nat. Commun. 12, 695 (2021). 33514707 10.1038/s41467-020-20698-9 PMC7846611

[32] C. W. Lindeman, V. F. Hagh, C. I. Ip, S. R. Nagel, Competition between energy and dynamics in memory formation. Phys. Rev. Lett. 130, 197201 (2023). 37243648 10.1103/PhysRevLett.130.197201

[33] L. Xin, Y. Li, B. Wang, Z. Li, Magnetic poles enabled kirigami meta-structure for high-efficiency mechanical memory storage. Adv. Funct. Mater. 34, 2310969 (2024).

[34] O. R. Bilal, A. Foehr, C. Daraio, Bistable metamaterial for switching and cascading elastic vibrations. Proc. Natl. Acad. Sci. U.S.A. 114, 4603–4606 (2017). 28416663 10.1073/pnas.1618314114 PMC5422829

[35] X. Zhang, J. Ma, M. Li, Z. You, X. Wang, Y. Luo, K. Ma, Y. Chen, Kirigami-based metastructures with programmable multistability. Proc. Natl. Acad. Sci. U.S.A. 119, e2117649119 (2022). 35254898 10.1073/pnas.2117649119 PMC8931353

[36] S. Yi, L. Wang, Z. Chen, J. Wang, X. Song, P. Liu, Y. Zhang, Q. Luo, L. Peng, Z. Wu, C. F. Guo, L. Jiang, High-throughput fabrication of soft magneto-origami machines. Nat. Commun. 13, 4177 (2022). 35853940 10.1038/s41467-022-31900-5 PMC9296529

