# Supplementary material for: Stiffness reprogrammable magnetorheological metamaterials inspired by spine for multibit visual mechanical information processing
Source: Sci Adv. 2025 Oct 8;11(41):eady8430. doi: 10.1126/sciadv.ady8430 (PMC12506972; doi:10.1126/sciadv.ady8430)
Supplement: Supplementary file 1 — Supplementary Text Figs. S1 to S21 Legends for movies S1 to S3 [file sciadv.ady8430_sm.pdf]

Science Advances
AAAS

Supplementary Materials for

Stiffness reprogrammable magnetorheological metamaterials inspired by spine for multibit visual mechanical information processing

Congcong Lou *et al.*

Corresponding author: Huaxia Deng, hxdeng@ustc.edu.cn; Xinglong Gong, gongxl@ustc.edu.cn

Sci. Adv. **11**, eady8430 (2025)
DOI: 10.1126/sciadv.ady8430

The PDF file includes:

Supplementary Text
Figs. S1 to S21
Legends for movies S1 to S3

Other Supplementary Material for this manuscript includes the following:

Movies S1 to S3

Supplementary Text

Supplementary Section 1: 3D printing of hard magnetic elastomer

The magnetorheological spine (MRS) beam is fabricated by 3D printing based on screw extrusion. The preparation method for the powders used in printing is as follows (Fig. S1): the TPR 30A particles are first frozen through liquid nitrogen, then quickly ground with an open mill. This step is repeated until the particles are ground to a powder with a diameter of less than 2 mm. Then, the TPR 30A powders are evenly mixed with the same weight NdFeB particles at 50 °C, allowing the NdFeB particles to coat the surface of the TPR 30A powders. The mixed powders are then poured into the screw extruder to obtain the magnetic elastomer wires (TPR 30A/NdFeB 50 wt%). To improve dispersion uniformity, these segments undergo multiple re-extrusion cycles. This reprocessing step helps break up any particle agglomerates and promotes a more homogeneous distribution of NdFeB particles throughout the TPR 30A matrix. Finally, the magnetic wires are frozen in liquid nitrogen and ground to obtain the composite hard magnetic powders with a diameter of 2 mm. The MRS beam is printed by a home-made screw printing device. The nozzle diameter is 0.4 mm, the height of each layer is 0.25 mm, and the filling density is set to 100%. The print speed is 15 mm/s, and the nozzle temperature is set to 160 ℃. The magnetic powder is transferred to the heating area through the rotation of the screw, melted and extruded from the nozzle. When the molten hard-magnetic elastomer is extruded, it is quickly cooled and solidified at room temperature to provide support for the next layer of printing. The superposition of multiple layers realizes the printing of three-dimensional samples.

The key innovation of our screw extrusion-based 3D printing method lies in the material feeding strategy. Unlike conventional fused deposition modeling (FDM), which relies on gear-driven filament feeding, our method enables the use of soft composite materials by converting them into powder form and feeding them via a rotating screw. Traditional FDM systems often encounter filament buckling and feeding failure when printing soft materials, which limits their applicability. Our screw extrusion design effectively overcomes this constraint, substantially expanding the range of printable materials to those with lower modulus. Regarding printing precision, the resolution of our screw extrusion system is primarily determined by the same factors as traditional FDM: the mechanical precision of the motion control system (stepper motors and linear guides) and the nozzle diameter. In our setup, the typical printing accuracy is approximately 0.1 mm.

The microstructure of the materials can be observed by SEM. The microstructure of the NdFeB particles was shown in Fig. S2a, with an average particles size of about 7 microns. To confirm the uniformity of the dispersion, we have conducted SEM analysis of both the powdered form and the printed samples. The SEM images demonstrate that the NdFeB particles are uniformly distributed in both states, validating the effectiveness of our processing steps in achieving a well-dispersed mixture (Fig. S2b). The surface of the printed sample is smooth, with the high printing precision (Fig. S2c). The uniform distribution of NdFeB particles in the TPR 30A matrix improves due to the heating and extrusion process in the nozzle. Moreover, the uniform distribution of NdFeB particles ensures the deformation of the MRS beam under the magnetic field (Fig. S2d).

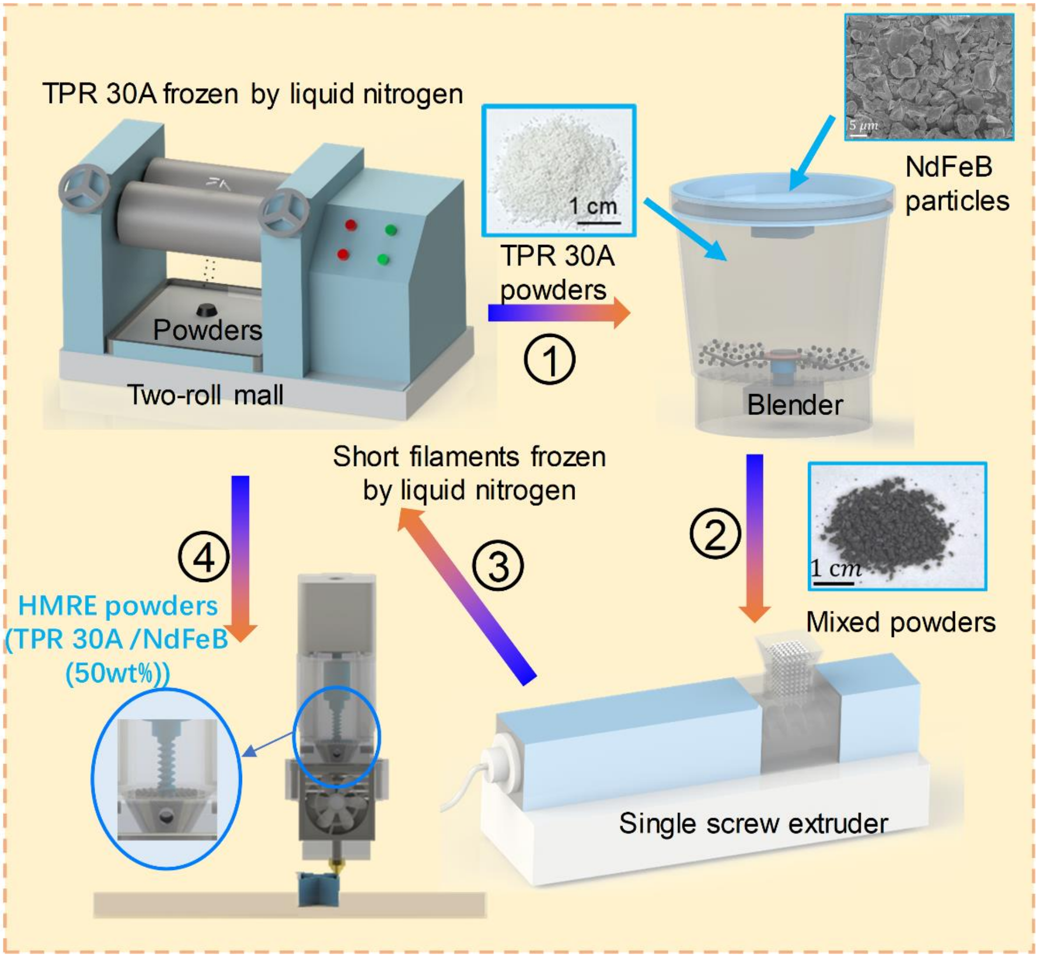

Fig. S1. The preparation process of HRME powders and the printing processing.

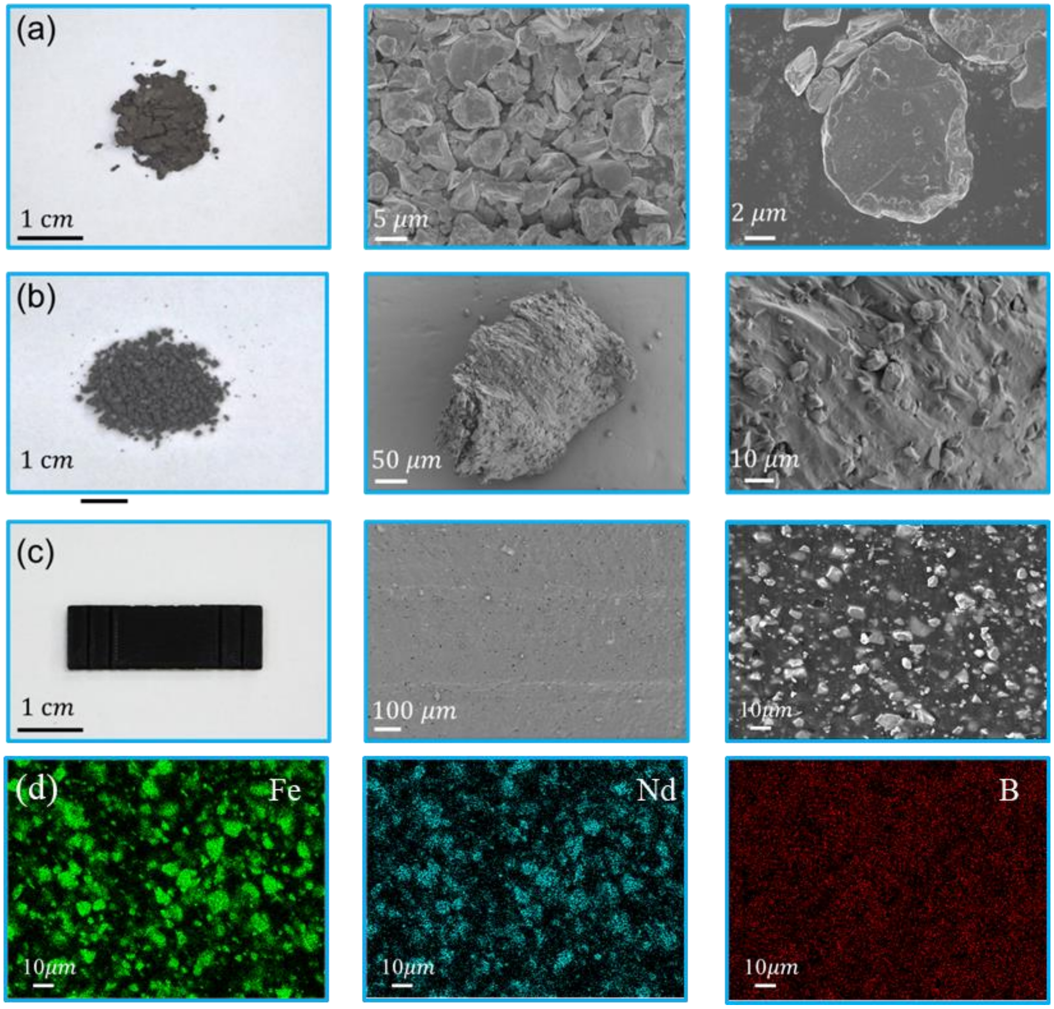

Fig. S2. SEM of printed material. a) The microscopic size of NdFeB particles. b) The microstructure of the printed powders. c) The microstructure of the print sample surface. d) Fe-, Nd-, B-element mappings of the printed sample.

Supplementary Section 2: The stiffness variation of MRS beams with different geometric parameters

Firstly, we study the mechanical performance of the MRS beam, the fracture experiment is conducted (Fig. S3). The tensile rate is 0.1 mm/s. The fracture stress is 0.83 MPa and the fracture strain is about 150%. Then, the compression experiments are conducted on MRS beams with different thicknesses (Fig. S4). During the compression process, the MRS beam in 'ON' state presents a positive stiffness, while the MRS beam in 'OFF' state presents a negative stiffness. The negative stiffness of the MRS beam in 'OFF' state is closely related to the thickness, and the negative stiffness increases sharply with the increase of the thickness. While the positive stiffness of the MRS beam in 'ON' state mainly depends on the contact of the gaps, and the stiffness increases slightly with the increase of thickness.

To study the effect of gap width on the stiffness of MRS beams in two stable states, compression experiments of MRS beams with different gap widths were conducted (Fig. S5). With the increase of the gap width, the tightness of the contact between the two sides of the gap decreases, resulting in the substantial stiffness reduction of MRS beam in the 'ON' state. However, for the MRS beam in the 'OFF' state, the negative stiffness is not sensitive to the gap width. With the increase of the gap width, the beam length increases slightly, resulting in a slight decrease in negative stiffness.
The effect of gap height on the stiffness of MRS beams in two stable states is studied based on the compression experiments of MRS beams with different gap height (Fig. S6). With the increase of gap height, of the gap contact is tighter under the same compression displacement in the 'ON' state, resulting in the increase of stiffness. As for the MRS beam in 'OFF' state, the increase of gap height has little effect on its deformation. Thus, with the increase of gap height, the negative stiffness of MRS beam in the 'OFF' state hardly changes.

The effect of length on the stiffness of MRS beams in two stable states is also studied (Fig. S7). Here, the compression experiments of MRS beams with different length are conducted. With the increase of the length, the stiffness substantially decreases in both two stable states.

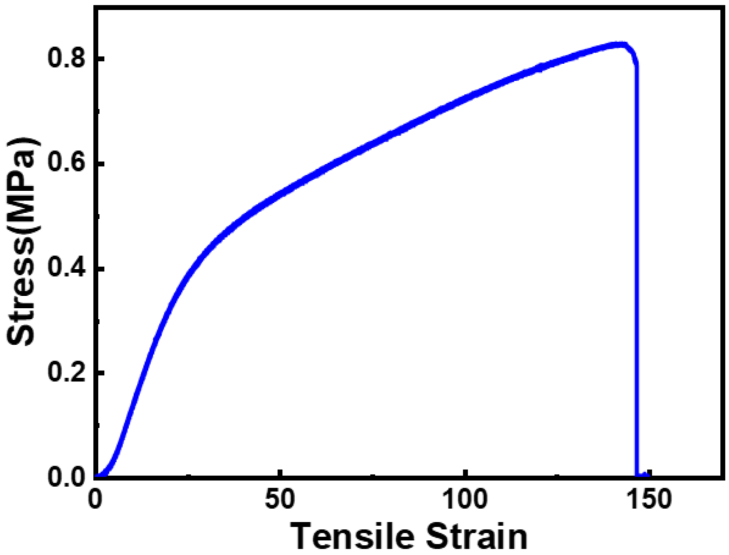

Fig. S3. The tensile fracture test of MRS beam.

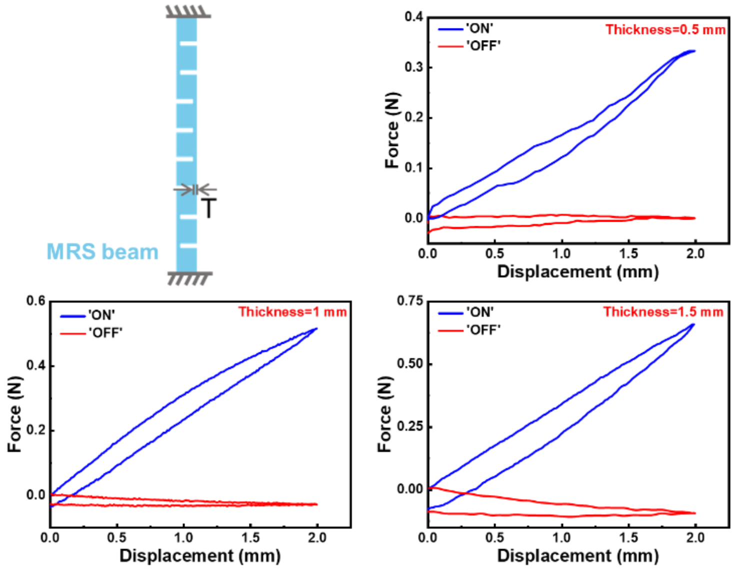

Fig. S4. Compression displacement curves of MRS beam under two states with different thicknesses.

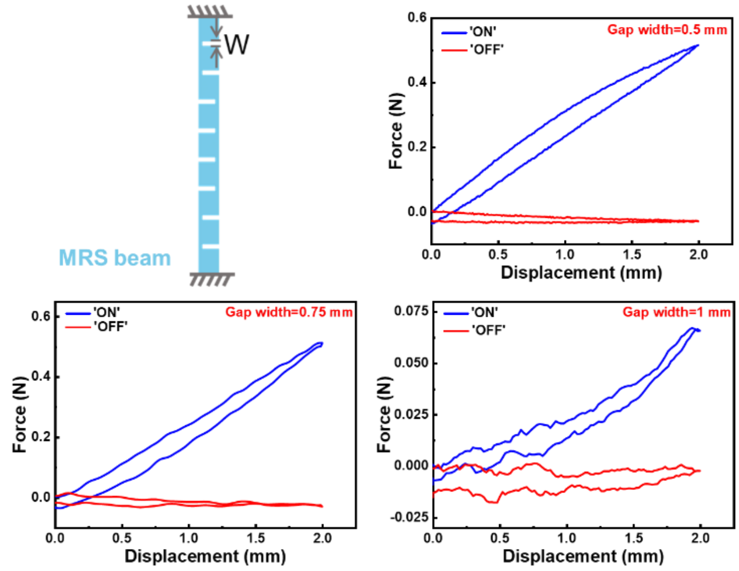

Fig. S5. Compression displacement curves of MRS beam under two states with different gap width.

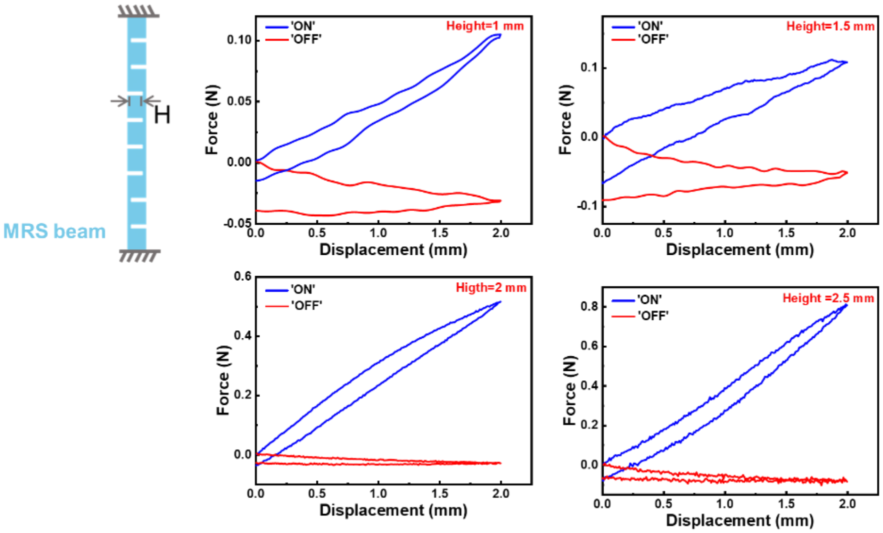

Fig. S6. Compression displacement curves of MRS beam under two states with different gap height.

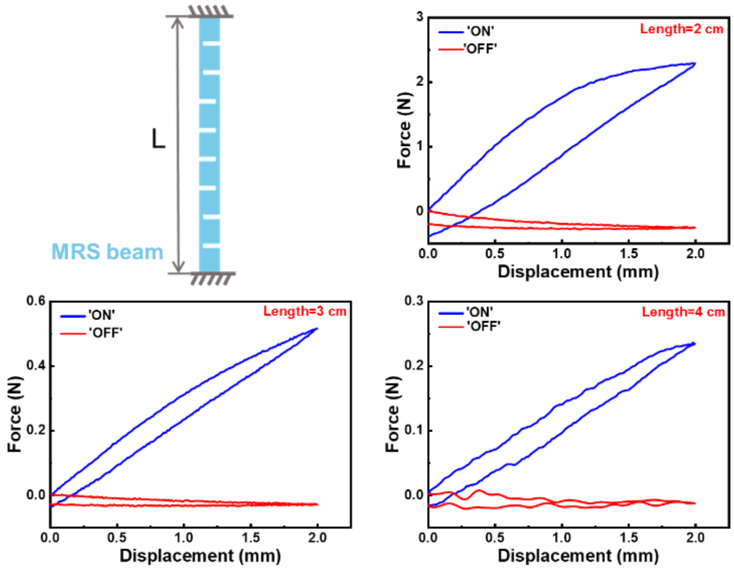

Fig. S7. Compression displacement curves of MRS beam under two states with different length.

Supplementary Section 3: The bi-stable stability test of MRS beams

To better understand the impact of hysteresis on the stability and functionality of the pixelated device, we have conducted additional cyclic experiments involving both the switching and loading/unloading behaviors of the MRS beams (Fig. S8). The cyclic axial compression experiments were conducted under constant displacement conditions (3 mm, 10 s cycle period) in both 'ON' and 'OFF' states. The results confirm that the beam maintains high stiffness in the 'ON' state and exhibits quasi-zero stiffness in the 'OFF' state, even after 1000 loading cycles. These findings further validate the long-term stability of the mechanical performance despite the presence of hysteresis.

Additionally, the cyclic magnetron switching experiment between two states of MRS beam is conducted (Fig. S9). The programmed power provides a periodic current of magnitude 2A, and a relay is utilized to convert direct current into an alternating current. Electromagnets provide alternating magnetic field with the input of current. MRS beam can switch between two states under the alternating magnetic field. The position of the middle point of the MRS beam is recorded by the laser displacement sensor.

Over 1000 cycles, the MRS beam demonstrated consistent switching behavior with negligible drift, indicating high magnetic reprogramming repeatability and mechanical stability. This suggests that the hysteresis observed in individual loading curves does not substantially impair device operation in dynamic applications.

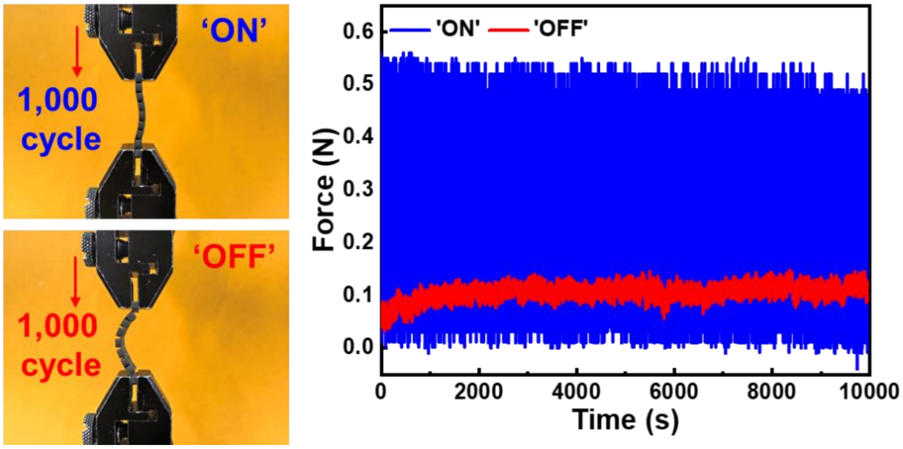

Fig. S8. The cyclic axial compression experiments of MRS beam in two states.

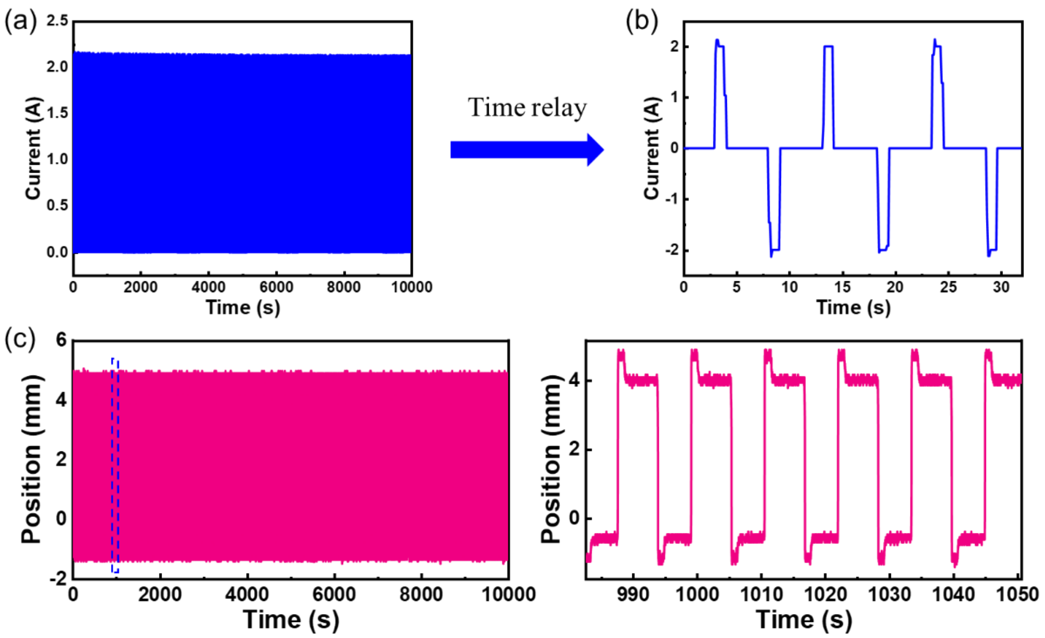

Fig. S9. The cyclic switching of the MRS beam between two states under programming magnetic field. a) The periodic 2 A current provided by a programmed power. b) The alternating current converted by a relay. c) The text position of the MRS beam under 1000 cycle transitions.

Supplementary Section 4: Magnetization and magnetically controlled deformation of MRS beam

The printed MRS beam is fixed in a ‘C’ shape and placed in a strong magnetic field for magnetization (Fig. S10). The magnetic field strength at the MRS beam surface (M_S) is test, which increases with the increase of external magnetic field strength. Furthermore, to obtain the optimal magnetization duration, the MRS beams are placed in a 1 T magnetic field for different durations and test its M_S. With the increase of magnetization duration, the magnetic field strength at the MRS beam surface increases. As a result, we put the MRS beam under a 1 T magnetic field for 15 minutes to magnetize it, enabling magnetic field-controlled bending behavior.

The mechanical performance of the MRS beam under different magnetic fields is studied (Fig. S11). First, we test the magnetic field distribution provided by electromagnets under different current inputs. Then, the compression tests of MRS beam are conducted under different magnetic fields. When the magnetic field increases lower than the required transition magnetic field, the MRS beam is in the ‘ON’ state, exhibiting a high stiffness. When the magnetic field increases higher than the required transition magnetic field, the state of the MRS beam switches to the ‘OFF’ state, presenting a lower stiffness.

The magnetic deformation of the MRS beam is simulated (Fig. S12). The initial state of the MRS beam is ‘ON’ state, when a uniform magnetic field is applied, the state of MRS beam changes from ‘ON’ to ‘OFF’ under the magnetic field.

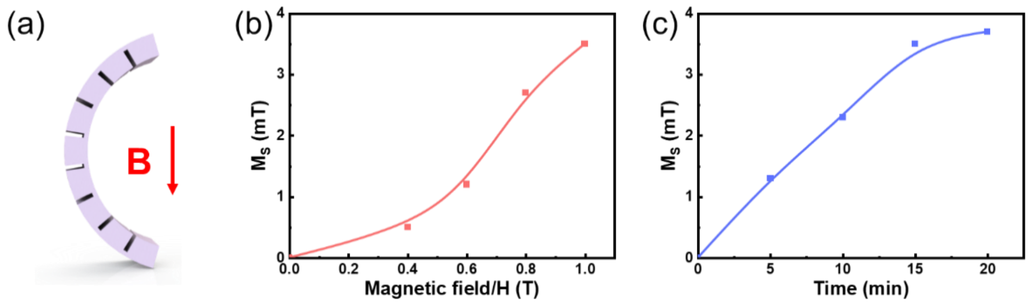

Fig. S10. The magnetization of the MRS beam. a) The shape of the MRS beam during magnetization process. b) The relationship between the magnetic field and the magnetic field strength at the beam surface (M_S). c) The relationship between magnetization time and M_S.

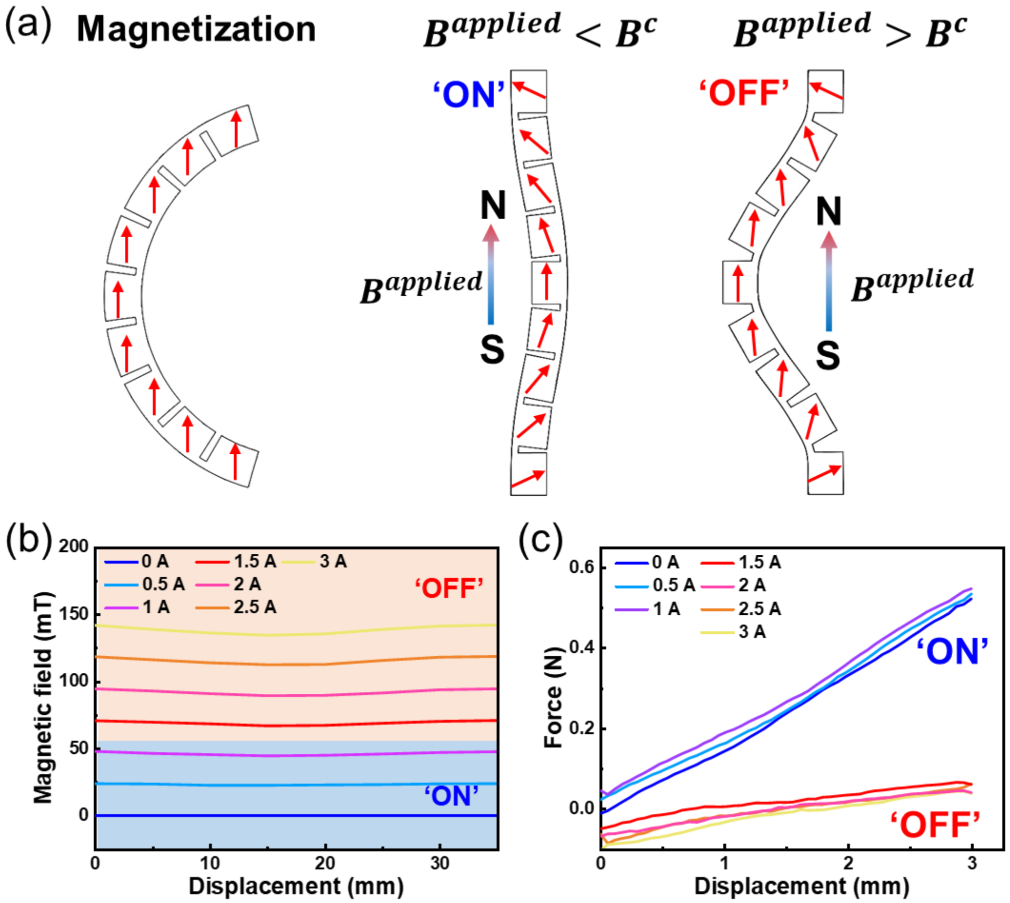

Fig. S11. Magnetic field effects analysis. a) The diagram of MRS beam deformation under different magnetic fields. b) The magnetic field distribution with different current inputs. c) The compression force-displacement curves of MRS beam under different magnetic fields.

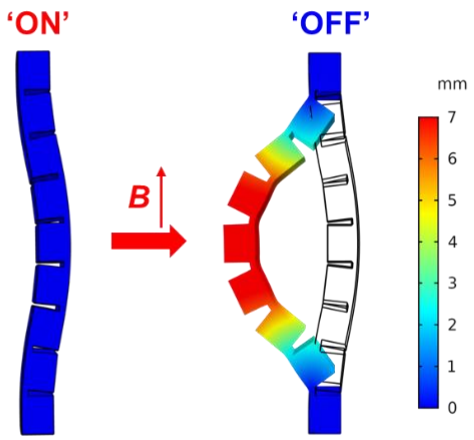

Fig. S12. The simulation of the MRS beam under nonuniform magnetic field.

Supplementary Section 5: Verification of the independence of stiffness coding and unit cell position

We conducted compression experiments on metamaterials with the same number of 'ON' state cells (ξ=1 and ξ=3) placed at different positions within the metamaterial structure. The results, as shown in Fig. S13, indicate that the stiffness of the metamaterial varies by less than 5% when the positions of the 'ON' state cells are altered. This minimal variation suggests that the stiffness is primarily determined by the number of 'ON' cells rather than their specific positions.

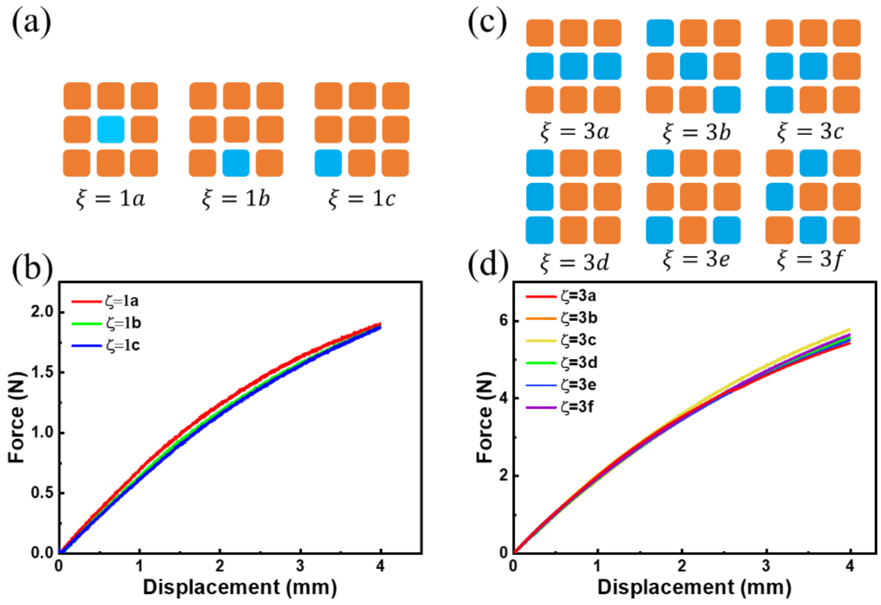

Fig. S13. The relation between the stiffness of metamaterial and the number and position of elements in ON state. a) The three options for the position of a metamaterial cell with only one 'ON' state cell. b) The compression displacement curves of metamaterials at three positions. c) The six options for the position of a metamaterial cell with three 'ON' state cells. d)The compression displacement curves of metamaterials at six positions.

Supplementary Section 6: The preparation process of force-induced luminescent materials and the luminescence principle of metamaterial encoding

The mechanoluminescent materials play an important role in the visualization of stiffness information. The preparation process of the mechanoluminescent material is as follows (Fig. S14). Firstly, ZnS:Cu particles are mixed with PDMS in the ratio of 7:3 and formed a uniform dispersion followed by adding the curing agent according to a PDMS/curing agent ratio of 10:1. Then, SiO_2 nanoparticles of 1.25% mass fraction of the total mixed solution are added and stirred for 5 min, and the mixed solution was put into a vacuum pump to evacuate the internal air bubbles. The above mixed solution is further added into the Acrylonitrile Butadiene Styrene plastic (ABS) mold and cured at 80 °C for 30 minutes to obtain mechanoluminescent films. The mechanoluminescent films show different intensity spots under different external forces.

To explore this relationship, we conducted experiments to evaluate the luminescence response under varying applied loads. The results demonstrate a nonlinear, positively correlated trend between light intensity and external load, thereby confirming the force-dependent nature of the mechanoluminescent response.

To address luminescence stability of the mechanoluminescent material, we performed cyclic stretching tests to evaluate the long-term performance under repeated mechanical loading (Fig. S16). The results, obtained after 1000 tensile cycles, demonstrate excellent luminescence stability, confirming the material's suitability for applications requiring repeated mechanical reprogramming and optical readout.

To further elucidate the luminescence mechanism of metamaterials, we draw the schematic of the binding of the mechanoluminescent material to the cell and the stress simulation of the mechanoluminescent material under external excitation when the cell is in different states. As shown in Fig. S17a, when the cell is in the 'ON' state, applying stimulus to the base of the cell, the high stiffness allows the force transfer to the mechanoluminescent material, causing light emission. In contrast, when the cell is in the 'OFF' state, the cell's quasi-zero stiffness absorbs the applied force internally, resulting in dark.

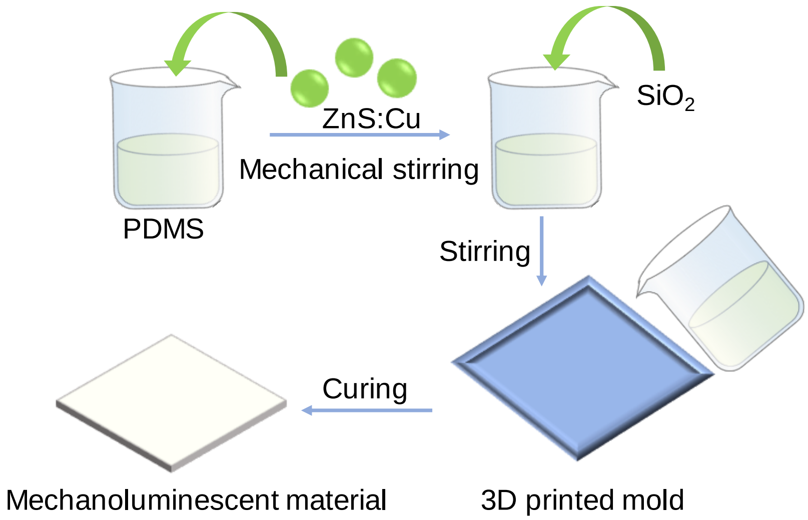

Fig. S14. The preparation process of the mechanoluminescent material.

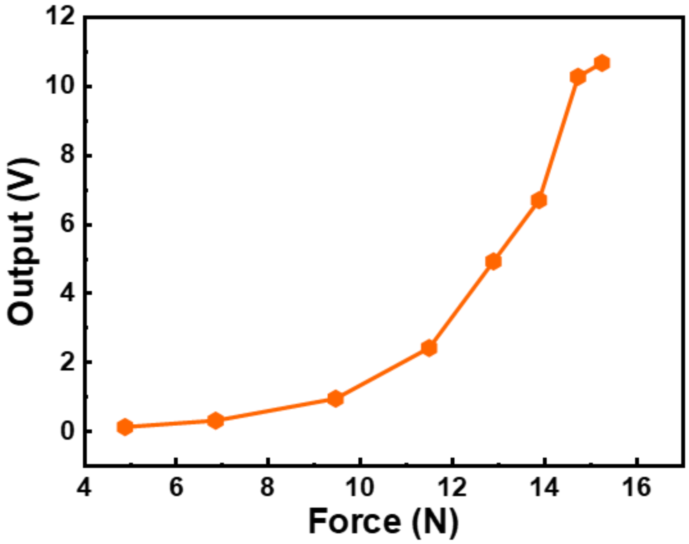

Fig. S15. The luminescence response of the mechanoluminescent material under varying strain rates.

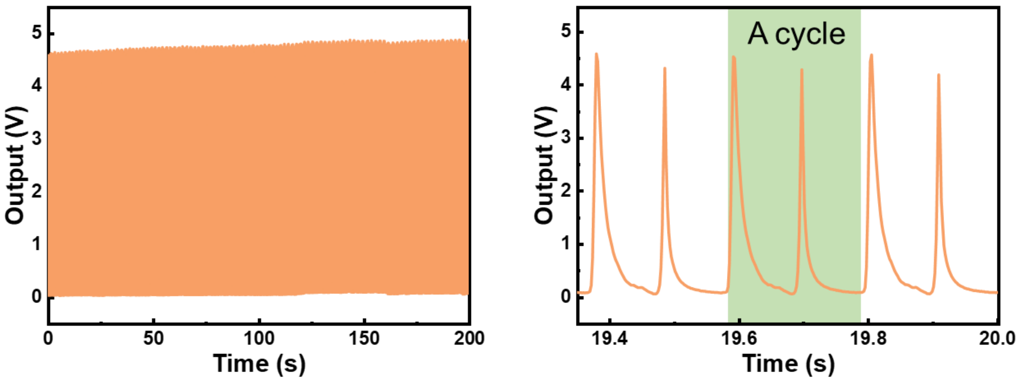

Fig. S16. The cyclic luminescence response of the mechanoluminescent material under tensile strain.

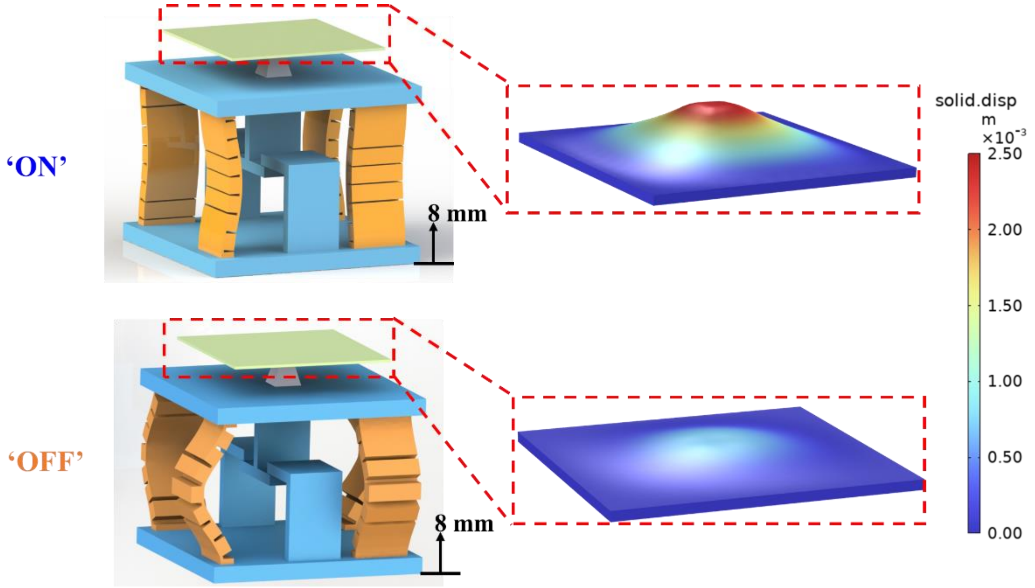

Fig. S17. The simulation of stress distribution of internal mechanoluminescent materials in mechanical metamaterial when the cell in a) 'ON' state or b) 'OFF' state under external excitation.

Supplementary Section 7: The OR logic gate operation of metamaterials

Here, a mechanical gate with an ‘OR’ logic function is built. The left and right series units (orange dotted box) are coupling controlled by magnetic field. The initial state of the mechanical gate is shown in Fig. S18a, all units are in the ‘OFF’ state. Regarding the determination of binary outputs (‘0’ or ‘1’) based on the force-displacement response: we established a quantitative threshold to classify the logic state. Specifically, if the output stiffness (slope of the force-displacement curve) exceeds three times the stiffness of a single cell in the ‘OFF’ state, the output is classified as ‘1’; otherwise, it is defined as ‘0’. Therefore, although the force-displacement curves of the second and fourth outputs in Fig. S18 (b) and (d) exhibit slight differences, both clearly exceed the threshold criterion and are thus correctly classified as logical ‘1’. When the displacement is applied above the mechanical gate (in= 0, 0), due to the low stiffness, no force is transmitted to the bottom (out= 0). As for in= (0, 1), the right series element is transformed to the ‘ON’ state by magnetic field regulation. Applying the same displacement, the force can be transferred due to the stiffness transformation. Similarly, when in= (1, 0) or (1, 1), the force also can be transferred due to the stiffness transformation.

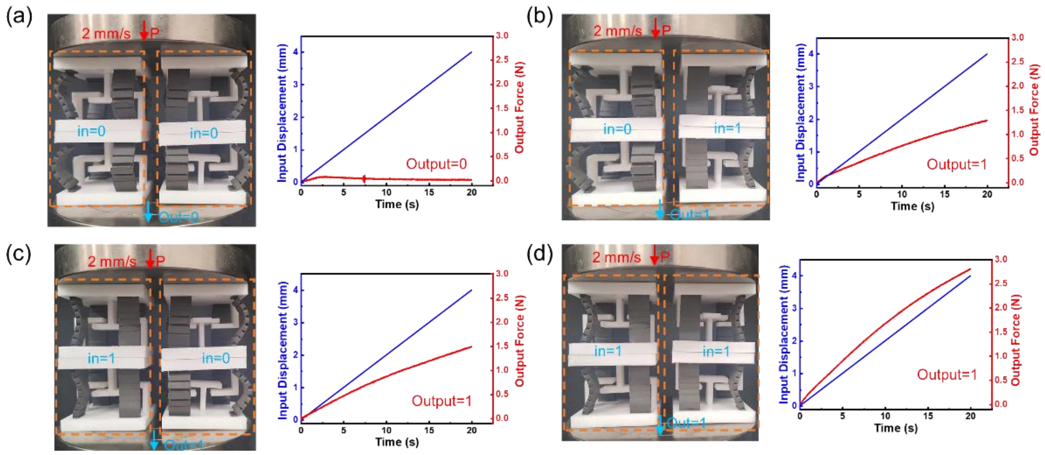

Fig. S18. The 'OR' mechanical logic computation gate. a) Input (0, 0), Output (0). b) Input (0, 1), Output (1). c) Input (1, 0), Output (1). d) Input (1, 1), Output (1).

Supplementary Section 8: The theoretical analysis of compression and axial force in 'ON' and 'OFF' states

To analyze the stiffness difference of the imitation spinal beam in 'ON' and 'OFF' states, the simplified model is established (Fig. S19). When the beam in the 'ON' state, the gaps touch each other during pre-compression, it can be simplified to a solid curved beam. For the beam in the 'OFF' state, the bulges between adjacent gaps can be ignored, the beam is simplified to a thin-walled curved beam.

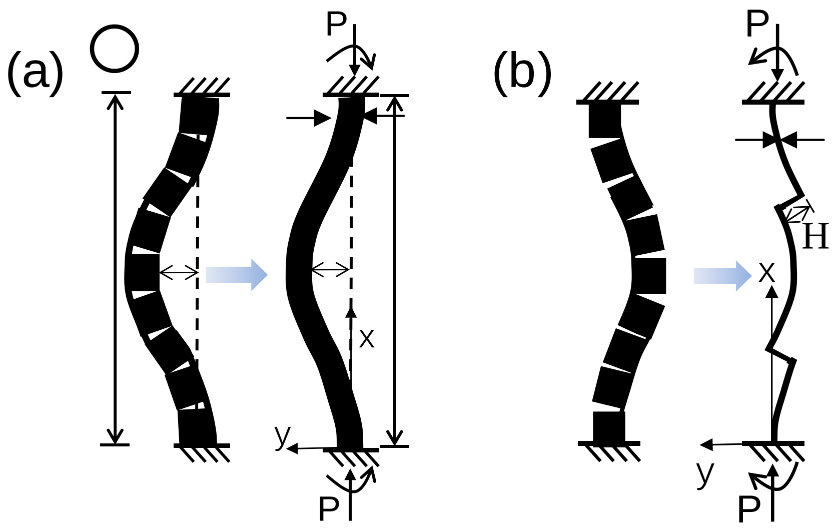

Fig. S19. The simplified model of imitation spinal beam in a) 'ON' state. b) 'ON' state.

The imitation spinal beam bends under pre-compression displacement, according to its shape characteristics, the shape curve can be assumed as

$$y = h(1 - \cos(\frac{2\pi x}{}))$$ (1)

The total length of the beam is L_0, which can be regarded as a constant during bending.

$$L_0 = \int^{l} \sqrt{1 + \left(\frac{dw}{dx}\right)^2}\, dx \approx \int^{l} 1 + \frac{1}{2}\left(\frac{dw}{dx}\right)^2 dx$$ (2)

The relationship between the height of the beam and the amplitude of the beam shape can be obtained.

$$= \sqrt{\frac{l(L_0 - l)}{\pi^2}}$$ (3)

For the imitation spinal in the 'ON' state, the initial shape can be assumed to be:

$$y = h_0(1 - \frac{\cos 2\pi x}{}) \qquad (4)$$

According to the equation of the deflection and moment:

$$\frac{d^2 w}{dx^2} = \frac{}{EI} \qquad (5)$$

where E is the young's modulus of material, $I_{ON} = \frac{\bar{b}\bar{h}_{ON}^{\ 3}}{12}$, the width of the beam $\bar{b} = 10$ mm, the thickness $\bar{h}_{ON} = 2.5$ mm. The beam bends under axial load, the shape is still a cosine function, and the deflection curve can be obtained

$$w = (h - h_0)(1 - \frac{\cos 2\pi x}{}) \qquad (6)$$

The moment at different positions consists of the moment at the constrain and the moment generated by the axial load.

$$M = -\left(M_0 + P(y + w)\right) \qquad (7)$$

Substitute equations (6) and (7) into equation (5) and take the derivative

$$EI\left((h - h_0)\left(\frac{2\pi}{}\right)^3 \sin\left(\frac{2\pi x}{}\right)\right) = \frac{Ph2\pi}{} \sin(\frac{2\pi x}{})$$

The relation between the axial load P_{ON} and the height *l* of the beam at bending is as follows:

$$P_{ON} = \frac{EI_{ON}(h - h_0)\left(\frac{2\pi}{}\right)^2}{}$$

Similarly, for the imitation spinal beam in the 'OFF' state, the initial shape of the simplified beam can be assumed as:

$$y = \begin{cases} \left(1 - \frac{\cos 2\pi x}{}\right) & \left(0 < \frac{x}{} < \frac{a}{L}\right) \\ \left(1 - \frac{\cos 2\pi x}{}\right) - H & \left(\frac{a}{L} < \frac{x}{} < 1 - \frac{a}{L}\right) \\ \left(1 - \frac{\cos 2\pi x}{}\right) & \left(1 - \frac{a}{L} < \frac{x}{} < 1\right) \end{cases}$$

The relation between the axial load *P*   and the height *l* of the beam at bending can be obtained as follows:

$$P \quad = \frac{EI_{OFF}(h - h_0)\left(\frac{2\pi}{}\right)^2}{}$$

where $I_{OFF} = \frac{\bar{b}\bar{h}_{OFF}^{3}}{12}$, $\bar{b} = 10$ mm, $\bar{h}_{OFF} = 0.5$ mm. The theoretical result of the compression and axial force is shown in the Fig. S20. In 'ON' state, the beam shows a higher stiffness, while in the 'OFF' state, the stiffness of the beam is closed to zero.

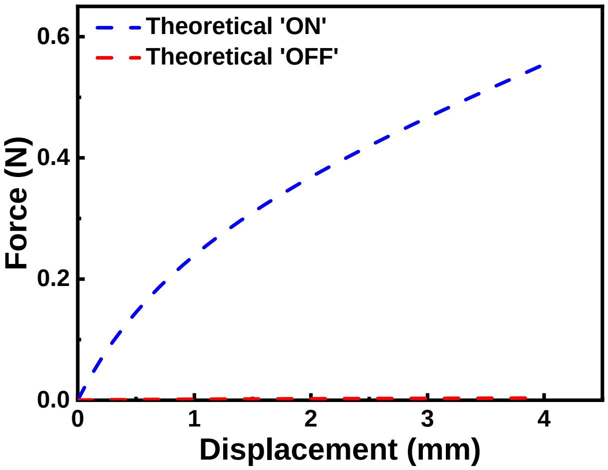

Fig. S20. The theoretical result of the compression and axial force of the beam in 'ON' and 'OFF' state.

Supplementary Section 9: The theoretical analysis of mechanical performance of HMRE in nonuniform magnetic field.

In Section 8, the axial load P can be expressed as

$$\begin{cases} P_{ON} = \dfrac{EI_{ON}(h-h_0)\left(\frac{2\pi}{}\right)^2}{} \\ P = \dfrac{EI_{OFF}(h-h_0)\left(\frac{2\pi}{}\right)^2}{} \end{cases} \tag{1}$$

where $E = 2\boldsymbol{\sigma}/(\mathbf{F}\mathbf{F}^{\mathbf{T}} - \mathbf{I})$ is the equivalent modulus of HMRE, and $\boldsymbol{\sigma} = \boldsymbol{\sigma}^{elastic} + \boldsymbol{\sigma}^{\boldsymbol{magnetic}}$, which is related to the external magnetic field. To study the relationship of E and applying magnetic field $\boldsymbol{B}^{applied}$, we established the force-magnetic model.

Consider a deformable HMRE in the reference state with material particles labeled by their position vectors **X**. The material particle **X** occupies the position dictated by a smooth deformation map: $\mathbf{x} = \chi(\mathbf{X})$. The deformation gradient tensor **F** is defined by

$$\mathbf{F} = \mathrm{Grad}\,\chi \tag{2}$$

where Grad denotes the gradient operator with respect to **X**. As conventional, we further denote by $J = \det(\mathbf{F})$, the deformation Jacobian.

We denote the magnetic flux density vector in the current configuration and the reference configuration of the considered material as $\boldsymbol{B}$ and $\widetilde{\boldsymbol{B}}$, standard kinematics can be used to express the relation between $\boldsymbol{B}$ and $\widetilde{\boldsymbol{B}}$ as

$$\widetilde{\boldsymbol{B}} = J\mathbf{F}^{-1}\boldsymbol{B} \tag{3}$$

We can define the magnetic potential energy, or the magnetic part of the Helmholtz free energy, per unit volume in the current configuration as the work required to realign the magnetic moment $\mu_0^{-1}\mathbf{M}$ along the applied magnetic field $\boldsymbol{B}^{applied}$ as follows:

$$W^{\mathrm{magnetic}} = -\frac{1}{\mu}\mathbf{M}\cdot\boldsymbol{B}^{applied} \tag{4}$$

Where μ_0 is the permeability of the HMRE, **M** is the magnetization density of the HMRE and the applied nonuniform magnetic field can be expressed as $\boldsymbol{B}^{applied} = \boldsymbol{B}_{\mathbf{0}} + \Delta\boldsymbol{B}$. From the relation $\widetilde{W}^{\mathrm{magnetic}} = W^{\mathrm{magnetic}}J$ and Eq. (3), the magnetic Helmholtz free energy per unit reference volume can be expressed as

$$\widetilde{W}^{\mathrm{magnetic}} = -\frac{1}{\mu}\mathbf{F}\widetilde{\mathbf{M}}\cdot\boldsymbol{B}^{applied} \tag{5}$$

Overall, the combined total Helmholtz free energy of the ideal hard-magnetic soft material per unit volume in the reference configuration can be expressed as

$$\widetilde{W} = \widetilde{W}^{\text{elastic}} + \widetilde{W}^{\text{magnetic}} \tag{6}$$

The nominal Helmholtz free energy density $\widetilde{W}$ is regarded as a function of two independent variables $\mathbf{F}$ and $\widetilde{\boldsymbol{B}}$ with one state parameter $\widetilde{\mathbf{M}}$. By work conjugation, we can obtain the Cauchy stress as:

$$\begin{aligned}\boldsymbol{\sigma} &= \frac{1}{J}\frac{\partial \widetilde{W}\left(\mathbf{F}, \widetilde{\boldsymbol{B}}\right)}{\partial \mathbf{F}}\mathbf{F}^{\mathrm{T}} \\ &= \frac{1}{J}\frac{\partial \widetilde{W}^{\text{elastic}}(\mathbf{F})}{\partial \mathbf{F}}\mathbf{F}^{\mathrm{T}} - \frac{1}{\mu}\boldsymbol{B}^{applied} \otimes \mathbf{M} - \frac{1}{\mu}\mathbf{F}\widetilde{\mathbf{M}} \cdot \frac{\partial \boldsymbol{B}^{applied}}{\partial \mathbf{F}}\end{aligned} \tag{7}$$

where $\widetilde{W}^{\text{elastic}}$ is a function of $\mathbf{F}$ only. Adopting the neo-Hookean constitutive model for the purely mechanical (elastic) response of the proposed material, the complete nominal Helmholtz free energy density function can be expressed as

$$\widetilde{W} = \frac{G}{2}\left(J^{-2/3}I_1 - 3\right) + \frac{K}{2}(J-1)^2 - \frac{1}{\mu}\mathbf{F}\widetilde{\mathbf{M}} \cdot \boldsymbol{B}^{applied} \tag{8}$$

where G is the shear modulus of the HMRE, K is the bulk modulus of the HMRE, and $I_1 = \mathrm{tr}(\mathbf{F}^{\mathrm{T}}\mathbf{F})$. From Eq. (7) the total Cauchy stress can be calculated as

$$\begin{aligned}\boldsymbol{\sigma} &= GJ^{-\frac{5}{3}}\left(\mathbf{F}\mathbf{F}^{T} - \frac{I_1}{3}\mathbf{I}\right) + K(J-1)\mathbf{I} \\ &- \frac{1}{\mu_0 J}(\boldsymbol{B}_{\mathbf{0}} + \Delta\boldsymbol{B}) \otimes \mathbf{F}\widetilde{\mathbf{M}} - \frac{1}{\mu}\mathbf{F}\widetilde{\mathbf{M}} \cdot \frac{\partial \Delta\boldsymbol{B}}{\partial \mathbf{F}}\end{aligned} \tag{9}$$

with its elastic and magnetic contributions expressed as

$$\boldsymbol{\sigma}^{elastic} = GJ^{-5/3}\left(\mathbf{F}\mathbf{F}^{T} - \frac{I_1}{3}\mathbf{I}\right) + K(J-1)\mathbf{I} \tag{10}$$

$$\boldsymbol{\sigma}^{magnetic} = -\frac{1}{\mu}(\boldsymbol{B}_{\mathbf{0}} + \Delta\boldsymbol{B}) \otimes \mathbf{F}\mathbf{M} - \frac{1}{\mu}\mathbf{F}\widetilde{\mathbf{M}} \cdot \frac{\partial \Delta\boldsymbol{B}}{\partial \mathbf{F}} \tag{11}$$

Therefore, the equivalent modulus of the MRS beam can be expressed as $E = \boldsymbol{\sigma}/\mathbf{F}$.

In this work, the magnetization density $\mathbf{M}$ is as low as 5 mT and the nonuniformity of the applied magnetic field is kept within 6 %, thus, the $\boldsymbol{\sigma}^{magnetic}$ is much lower than the $\boldsymbol{\sigma}^{elastic}$ and can be ignored.

Supplementary Section 10: The theoretical analysis of switching of bi-stable MRS beam between two states under lateral load *f*.

In order to simplify the calculation, the MRS beam is simplified as a straight beam. When the MRS beam is subjected to axial load p, the beam equation can be expressed as

$$EI\frac{d^4w}{dx^4} + p\frac{d^2w}{dx^2} = 0 \quad (1)$$

where *w* is the lateral beam displacement, *E* is the Young's modulus of the beam material and *I* is the moment of inertia of the beam. With clamped-clamped conditions, the boundary conditions to (1) are

$$w(0) = w(l) = 0, \left(\frac{dw}{dx}\right)_{x=0} = \left(\frac{dw}{dx}\right)_{x=l} = 0 \quad (2)$$

Normalize the axial force p by

$$N^2 = \frac{pl^2}{EI} \quad (3)$$

In order to have nonzero solutions, N must satisfy

$$\sin\left(\frac{N}{2}\right)\left[\tan\left(\frac{N}{2}\right) - \frac{N}{2}\right] = 0 \quad (4)$$

which allows two kinds of solution, when $j = 1, 3, 5, \ldots$

$$w_j(x) = C\left[1 - \cos\left(N_j\frac{x}{\ }\right)\right]$$
$$N_j = (j+1)\pi \quad (5)$$

and when $\mathrm{j} = 2, 4, 6, \ldots$

$$w_j(x) = C[1 - 2\frac{x}{\ } - \cos\left(N_j\frac{x}{\ }\right) + \frac{2\sin\left(N_j\frac{x}{\ }\right)}{N_j}]$$
$$N_j = 2.86\pi, 4.92\pi, \ldots \quad (6)$$

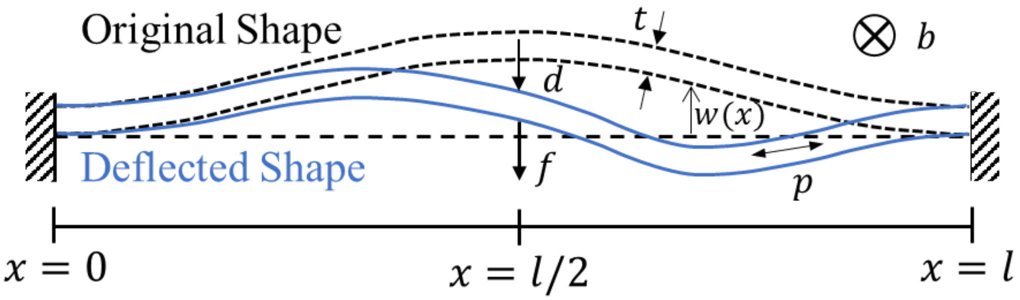

Fig. S21. Geometry and notation for curved beam deflection analysis.

Consider the single beam shown in Fig. S21. It has thickness *t*, depth *b*, span *l*, Young's modulus *E* and moment of inertia *I*. Let $w(x)$ denote the distance of the beam from the straight line connecting its two boundaries. The as-fabricated shape of the beam is

$$\overline{w}(x) = \frac{\ }{2}[1 - \cos(2\pi\frac{x}{\ })] \quad (7)$$

where *h* is the initial apex height of the beam. It will be explained later that having such an initial shape is one of several requirements for a curved beam to be bi-stable. Also as can be seen later in

the modeling, the geometry constant Q is critical to the behavior of this mechanism, and it is defined as

$$Q = \frac{}{t} \tag{8}$$

As the lateral force f is applied to the center of the beam at $x = l/2$, the center of the beam deflects by

$$d = \bar{w}\left(\frac{}{2}\right) - w\left(\frac{}{2}\right) \tag{9}$$

The total length of the beam s changes to

$$\text{s} = \int^{l} \sqrt{1 + \left(\frac{dw}{ds}\right)^{2}}\, dx \approx \int^{l} [1 + \frac{1}{2}\left(\frac{dw}{dx}\right)^{2}]\, dx \tag{10}$$

where a small deflection assumption is used. The change s of gives rise to the axial force p by Hooke's law such that

$$p = Ebt\left(1 - \frac{s}{(s)_{w=\bar{w}}}\right) \tag{11}$$

During deflection, define u_b as the beam bending energy, u_s as the beam compression energy, and u_f as the actuation energy. The variation of the bending energy inside the beam is

$$\partial(u_b) = \partial[\frac{EI}{2}\int^{l}\left(\frac{d^2\bar{w}}{dx^2} - \frac{d^2w}{dx^2}\right)^{2} dx] \tag{12}$$

The variation of the compression energy inside the beam is

$$\partial(u_s) = -p\,\partial(\text{s}) \tag{13}$$

The variation of the actuation energy is

$$\partial(u_f) = -f\,\partial(d) \tag{14}$$

A mode superposition method is used to solve the beam deflection. To simplify the superposition, first normalize the parameters according to

$$X = \frac{x}{}, W(X) = \frac{w(Xl)}{} \tag{15}$$

Next, normalize the applied force and parameters in (9)-(14) according to

$$F = \frac{fl^3}{EIh}, \Delta = \frac{d}{h}, S = \frac{sl}{h^2}, U_b = \frac{u_b l^3}{EIh^2}, U_s = \frac{u_s l^3}{EIh^3}, U_f = \frac{u_f l^3}{EIh^2} \tag{16}$$

When the energy is minimum and required to be greater than or equal to zero, ignoring the higher order term can be obtained

$$F = \frac{3\pi^4 Q^2}{2}\Delta\left(\Delta - \frac{3}{2} + \sqrt{\frac{1}{4} - \frac{4}{3Q^2}}\right)\left(\Delta - \frac{3}{2} - \sqrt{\frac{1}{4} - \frac{4}{3Q^2}}\right) \tag{17}$$

Movie S1. Bi-stable state transition process.

This movie presents the bi-stable state transition of cell in SPMM under external magnetic field.

Movie S2. Programmable information encoding.

This movie presents the programmable information encoding function of SPMM under external magnetic field, such as ‘U’, ‘S’, ‘T’, ‘C’.

Movie S3. Logic gate operation.

This movie presents the ‘OR’ mechanical logic computation gate.
